# Alpha-enolase regulates the malignant phenotype of pulmonary artery smooth muscle cells via the AMPK-Akt pathway

**DOI:** 10.1038/s41467-018-06376-x

**Published:** 2018-09-21

**Authors:** Jingbo Dai, Qiyuan Zhou, Jiwang Chen, Megan L. Rexius-Hall, Jalees Rehman, Guofei Zhou

**Affiliations:** 1 Department of Pediatrics, University of Illinois at Chicago, Chicago, IL 60612 USA; 2 Division of Pulmonary, Critical Care Medicine, Sleep and Allergy, Department of Medicine, University of Illinois at Chicago, Chicago, IL 60612 USA; 3 Department of Bioengineering, University of Illinois at Chicago, Chicago, IL 60612 USA; 4 Division of Cardiology, Department of Medicine, University of Illinois at Chicago, Chicago, IL 60612 USA; 5 Department of Pharmacology, University of Illinois at Chicago, Chicago, IL 60612 USA; 6 State Key Laboratory of Respiratory Diseases, Guangzhou Institute of Respiratory Diseases, The First Affiliated Hospital of Guangzhou Medical University, Guangzhou, Guangdong, 510120 China

## Abstract

The molecular mechanisms underlying the metabolic shift toward increased glycolysis observed in pulmonary artery smooth muscle cells (PASMC) during the pathogenesis of pulmonary arterial hypertension (PAH) are not fully understood. Here we show that the glycolytic enzyme α-enolase (ENO1) regulates the metabolic reprogramming and malignant phenotype of PASMC. We show that ENO1 levels are elevated in patients with associated PAH and in animal models of hypoxic pulmonary hypertension (HPH). The silencing or inhibition of ENO1 decreases PASMC proliferation and de-differentiation, and induces PASMC apoptosis, whereas the overexpression of ENO1 promotes a synthetic, de- differentiated, and apoptotic-resistant phenotype via the AMPK-Akt pathway. The suppression of ENO1 prevents the hypoxia-induced metabolic shift from mitochondrial respiration to glycolysis in PASMC. Finally, we find that pharmacological inhibition of ENO1 reverses HPH in mice and rats, suggesting ENO1 as a regulator of pathogenic metabolic reprogramming in HPH.

## Abstract (web summary)

Metabolic reprogramming of smooth muscle cells (SMCs) plays an important role in the pathogenesis of pulmonary arterial hypertension (PAH). Here, Dai et al. show that the glycolytic enzyme alpha-enolase contributes to this reprogramming, and that its inhibition limits SMC proliferation and disease progression in animal models of PAH.

## Introduction

Pulmonary arterial hypertension (PAH) is a devastating cardiopulmonary disease characterized by a progressive increase in pulmonary vascular resistance and right ventricular failure, which is a critical cause of patient mortality^1^. Hyper-proliferation and resistance to apoptosis of pulmonary artery smooth muscle cells (PASMC) mirror a “malignant phenotype” seen in tumor cells and contribute to the pathophysiology of PAH^2^. PASMC from animal models of pulmonary hypertension (PH) and human tissues with PAH exhibit a consistent pattern of reprogrammed cellular metabolism, which closely aligns with the Warburg effect in cancers^3^. In these cells, mitochondrial glucose oxidation is suppressed, whereas glycolysis is upregulated as the major source of adenosine triphosphate production. The rapid metabolic turnover increases the biosynthesis, which is essential for cell proliferation and help the cells to avoid from apoptosis^4^.

The molecular mechanisms underlying this metabolic shift in PAH are incompletely understood. Enolase (ENO) is a metalloenzyme that catalyzes the dehydration of 2-phospho-d-glycerate (2-PG) to phosphoenolpyruvate (PEP) in the glycolytic pathway^5^. There are three isoforms of ENO, α, β, and γ; each is encoded by a separate gene. These isoforms form five different homodimers or heterodimers in cells. α-enolase (ENO1) is ubiquitous and has been detected in most tissues, whereas γ-enolase (ENO2) is expressed predominantly in nervous tissues and β-enolase (ENO3) mainly in skeletal muscle tissues^6^. Accumulating evidence has demonstrated that ENO1 is a multi-functional protein depending on its cellular localization^7,8^. Although the majority of ENO1 is cytosolic and promotes tumor pathogenesis and progression, ENO1 is also present on the cell surface as a plasminogen receptor to promote cell migration and cancer metastasis^9^. An alternative start codon translates *ENO1* into a 37-kDa protein named c-Myc promoter-binding protein (MBP-1), which localizes in the nucleus as a transcription repressor of *c-Myc*, and functions as a tumor suppressor^10^.

Several studies have reported ENO1-mediated metabolic reprogramming in cancer cells^11^. Silencing of ENO1 in cancer cells promotes the adaptation to catabolic pathways, restores acetyl-CoA bulk through enhanced β-oxidation, and fuels the tricarboxylic acid (TCA) cycle by the cataplerotic reactions of tyrosine and glutamine catabolism^12,13^. However, it remains unknown whether ENO participates in the metabolic reprogramming of PASMC in PAH. In our study, we show that expression levels of ENO1 are increased in the PASMC from patients with associated PAH (APAH) and in the lungs of rodent hypoxia- and Sugen/Hypoxia (SuHx)-induced PH models. The silencing or inhibition of ENO1 decreases PASMC proliferation, promotes differentiation, sensitizes them to apoptosis, and restores the mitochondrial respiration. Conversely, the overexpression of ENO1 in PASMC induces a synthetic, de-differentiated, anti-apoptosis, and glycolytic phenotype. We also show that ENO1 regulates PASMC phenotype changes via the AMPK-Akt pathway. Furthermore, the inhibition of ENO1 by AP-III-a4 (ENOblock) reverses hypoxia-induced PH in mice and SuHx-induced PH in rats. Therefore, our study provides evidence that ENO1 regulates metabolic reprogramming in PAH.

## Results

### ENO1 levels are elevated in hypertensive lungs

**Fig. 1:**
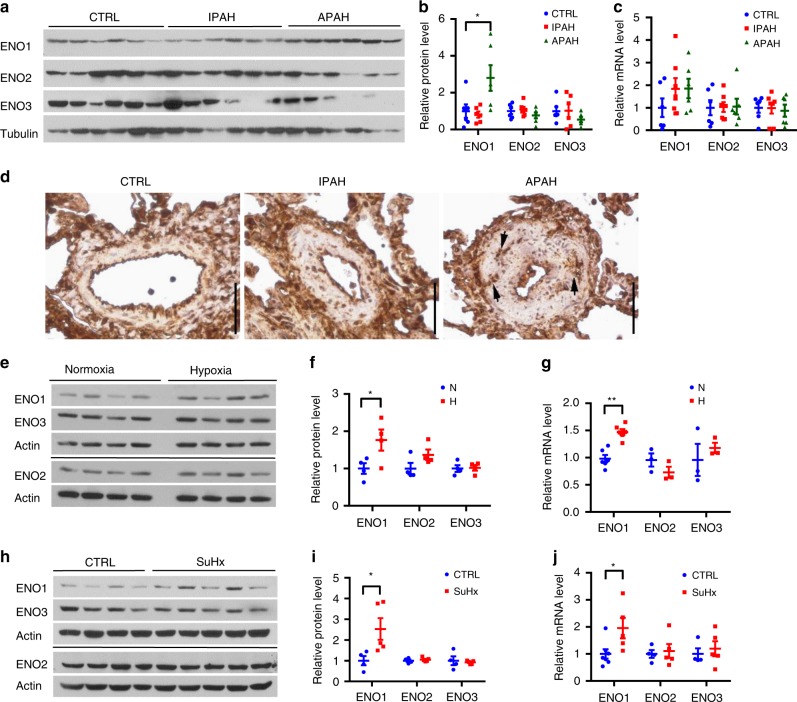
Increased expression levels of ENO1 in hypertensive APAH patients and experimental PH animal lungs. **a** PASMC isolated from patients with IPAH or APAH and control donors were lysed and subjected to western blotting to measure the level of enolases. **b** Normalized quantification of proteins demonstrates the expression level of ENO1, ENO2, and ENO3 in PASMC isolated from patients with IPAH or APAH and control donors (*n* = 6 per group, *P = 0.0223). **c** mRNA levels of ENO1, ENO2, and ENO3 in PASMC from patients with PAH and the control donors. **d** Immunohistochemistry of ENO1 in lung tissue sections of patients with PAH and the control donors. Brown color indicates the staining of ENO1. Black arrows indicate the elevated of ENO1 staining in the PASMC layer of the vessels (scale bars, 100 μm). **e** Whole lung tissues were isolated from hypoxia-induced PH mice and western blotting was used to measure the level of enolases. **f** Normalized quantification of proteins demonstrates the expression level of enolases in the whole lung tissue of hypoxia-induced PH mice (*n* = 4 per group, *P = 0.0263). **g** mRNA levels of enolase in the whole lung tissue of hypoxia-induced PH mice (*n* = 3–6, P < 0.001). **h** Whole lung tissues were isolated from SuHx-induced PH rats and western blotting was used to measure the level of enolases. **i** Normalized quantification of protein demonstrates the expression level of enolases in the whole lung tissue of SuHx-induced PH rats (*n* = 4–5 per group, *P = 0.0447). **j** mRNA levels of enolase in the whole lung tissue of SuHx-induced PH rats (*n* = 3–6, *P = 0.0303). Data represent the mean ± SEM. Student *t* test and one-way ANOVA were used to compare two and multiple groups. Bonferroni post-tests were carried out after ANOVA

We obtained human PASMC samples from normal donors, patients with idiopathic PAH (IPAH), and APAH from the Pulmonary Hypertension Breakthrough Initiative (PHBI)^14^. APAH samples include patients with Collagen Vascular Disease/Connective Tissue Disease and Congenital systemic-to-pulmonary shunts. Supplementary Table 1 shows the demographics, hemodynamic parameters, and 6-minute walk distance (6MWD) for PAH patients obtained from PHBI. We found that PASMC isolated from APAH patients but not from patients of IPAH, expressed markedly increased protein levels of ENO1 (Fig. 1a, b). However, there was no significant difference in ENO1 mRNA levels between IPAH and APAH PASMC and control donors (Fig. 1c). Both protein and mRNA levels of ENO2 or ENO3 remained unchanged between APAH/IPAH and control donors (Fig. 1a–c). ENO1 was ubiquitously expressed in the lung sections of PAH patients, including the media, intima, and the perivascular region, suggesting the expression of ENO1 in PASMC, endothelial cells, and monocyte/macrophages, however, it was mainly elevated in the media of the APAH patients (Fig. 1d). In experimental PH rodents, ENO1 was ubiquitously expressed in media, intima, and perivascular region and was significantly elevated in hypoxia-induced PH mice (Fig. 1e–g) and Sugen/hypoxia (SuHx)-induced PH rats (Fig. 1h-j), but not in the monocrotaline (MCT)-induced rat model of PH (Supplementary Fig. 1–2). Levels of ENO2 and ENO3 remained unchanged (Fig. 1e–j; Supplementary Fig. 1). As ENO1 catalyzes 2-PG to PEP in the glycolytic pathway^5^, we confirmed that hypoxic mice contained elevated PEP levels (Supplementary Fig. 3). These results suggest upregulation of ENO1 in PH.

### ENO1 promotes PASMC proliferation and de-differentiation

**Fig. 2:**
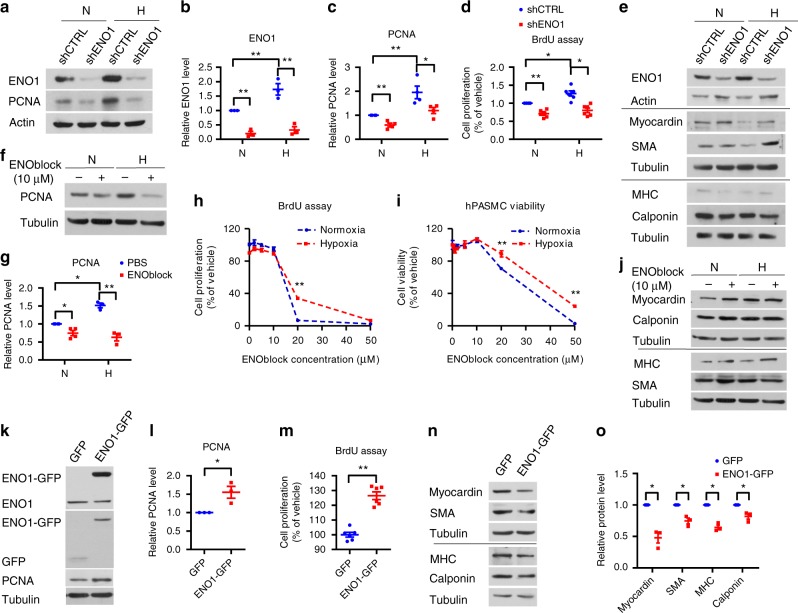
ENO1 promotes proliferation and suppresses expression of contractile proteins in PASMC. **a**–**c** Normal PASMCs were transfected with lentivirus containing shRNA targeting *ENO1* to silence *ENO1* (shENO1). The shENO1 and control (shCTRL) PASMC were treated with hypoxia (1% O_2_) for 24 h. We measured the expression level of ENO1 and PCNA by western blotting in cell lysates of shCTRL and shENO1–PASMC under normoxic and hypoxic conditions **a**. **b** shows the normalized quantification of ENO1 levels (*n* = 3 per group, **P < 0.01). **c** shows the normalized quantification of PCNA levels (*n* = 3 per group, *P < 0.05, **P < 0.01). **d** BrdU assay was used to measure the cell proliferation in shENO1–PASMC under normoxic and hypoxic conditions (*n* = 6, **P < 0.001). **e** Western blotting images of contractile proteins (Myocardin, SMA, MHC, and Calponin) in shCTRL and shENO1–PASMC under normoxic and hypoxic conditions. **f** PASMC were treated with 10 μM ENOblock and hypoxia (1% O2) for 24 h, and the PCNA levels were measured by western blotting. **g** Normalized quantification of PCNA level in ENOblock-treated PASMCs under normoxic and hypoxic conditions (*n* = 3–4 per group, *P < 0.05, **P < 0.01). **h** PASMCs were treated with ENOblock (in gradient concentrations) and hypoxia (1% O_2_) for 24 h, afterwards the cell proliferation (using BrdU assay) and **i** cell viability were measured (*n* = 4 per group, *P < 0.05, **P < 0.01). **j** PASMC were treated with 10 μM ENOblock and hypoxia (1% O_2_) for 24 h, the levels of SMC contractile proteins (Myocardin, Calponin, MHC, and SMA) were measured by western blotting. **k** PASMCs were transfected with pCMV3-ENO1-GFP. The transfection efficiency was determined by the detection of GFP and GFP-tagged ENO1 by GFP antibody and the overexpression of ENO1 was validated by ENO1 antibody (ENO1 and ENO1-GFP). The PCNA levels were also measured after overexpression of ENO1. **l** Normalized quantification of PCNA level in PASMC transfected with pCMV3-ENO1-GFP (*n* = 4 per group, *P = 0.0202). **m** BrdU assay of ENO1-overexpressed PASMC (*n* = 6 per group, **P < 0.0001). **n** Western blotting images and **o** normalized quantification of SMC contractile proteins (Myocardin, SMA, MHC, and Calponin) in ENO1-overexpressed PASMCs (*n* = 3 per group, *P < 0.05). Data represent the mean ± SEM. Student *t* test and one-way ANOVA were used to compare two and multiple groups. Bonferroni post-tests were carried out after ANOVA

During the development of PH, there is a PASMC phenotype switch from a differentiated state to a de-differentiated and proliferative state^15^. We silenced *ENO1* in PASMC with lentivirus encoding an shRNA that targets *ENO1* (shENO1) (Fig. 2a, b) and exposed them to normoxia and hypoxia (1% O_2_) for 24 h. Silencing of ENO1 significantly inhibited the expression of proliferating cell nuclear antigen (PCNA) and the bromodeoxyuridine (BrdU) incorporation (Fig. 2a–d), induced the expression of myocardin and α-smooth muscle actin (α-SMA), but did not alter the expression levels of myosin heavy chain (MHC) and calponin (Fig. 2e). To confirm that ENOblock, a newly identified small molecule ENO inhibitor, inhibits ENO activity in PASMC, we treated PASMC with 10 μM ENOblock for 8 h. ENOblock decreased ENO activity (Supplementary Fig. 4A) and PEP levels in PASMC exposed to normoxia or hypoxia, despite an elevated PEP levels during hypoxia (Supplementary Fig. 4B). ENOblock significantly inhibited PCNA levels (Fig. 2f, g), BrdU incorporation, and viability (Fig. 2h, i). However, hypoxic PASMC were more resistant to ENOblock (Fig. 2h, i). Treatment with ENOblock also induced the expression of myocardin, calponin, and MHC, but not α-SMA (Fig. 2j). These results suggest that ENO1 is necessary for PASMC proliferation and de-differentiation.

Conversely, overexpression of exogenous GFP-tagged ENO1 (Fig. 2k) significantly induced the PEP levels (Supplementary Fig. 5), PCNA levels (Fig. 2k, l), and BrdU incorporation (Fig. 2m), inhibited expression levels of myocardin, α-SMA, MHC, and calponin (Fig. 2n, o). Taken together, ENO1 is necessary and sufficient to promote PASMC proliferation and the synthetic, de-differentiated phenotype of PASMC.

### ENO1 induces PASMC resistance to apoptosis

**Fig. 3:**
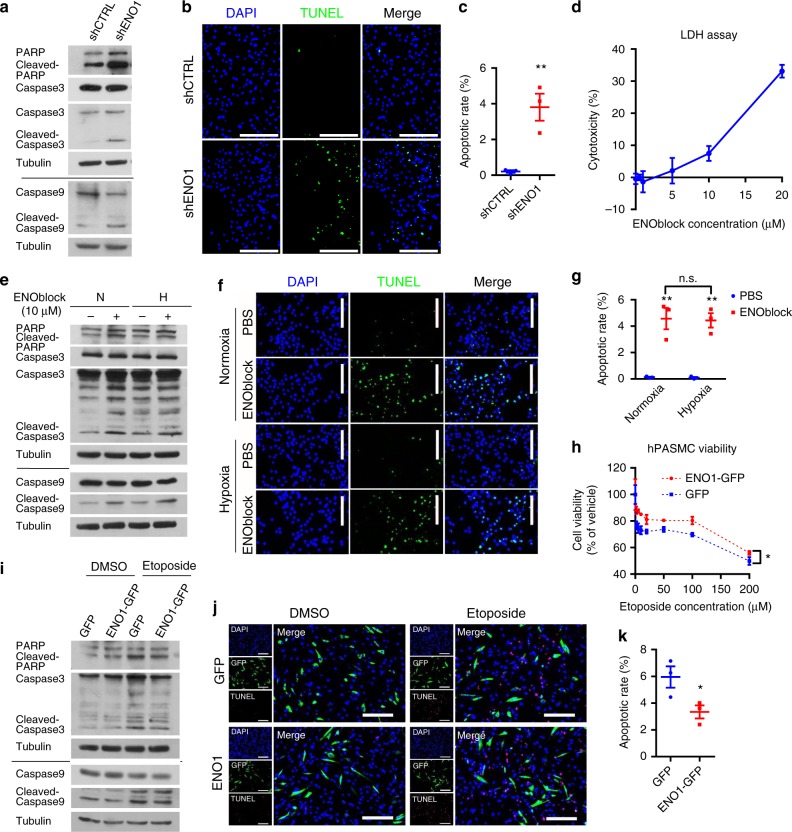
ENO1 induces PASMC resistance to apoptosis. **a** The amount of apoptotic markers: PARP (full length and cleaved), caspase 3 (full length and cleaved), caspase 9 (full length and cleaved) were detected by western blotting in shENO1–PASMC. **b** shENO1 and shCTRL PASMC were subjected to the TUNEL assay (Scale bars, 100 μm) and **c** the apoptotic rates were calculated (*n* = 3, **P < 0.0001) with the TUNEL assay in shENO1–PASMC. **d** PASMC were treated with ENOblock in different concentrations for 24 h, and the LDH assay was performed to detect cell death. **e** PASMCs were treated with 10 μM ENOblock and hypoxia (1% O_2_) for 24 h, and western blotting was used to detect apoptotic markers in cell lysate. **f** Images of the TUNEL assay (Scale bars, 100 μm) and **g** calculated apoptotic rates (*n* = 3 per group, **P < 0.0001) in ENOblock-treated PASMCs under normoxic and hypoxic conditions. **h** PASMCs were transfected with pCMV3-ENO1-GFP and treated with etoposide (in gradient concentrations) for 24 h. Afterwards, the cell viability was measured (*n* = 3 per group, *P < 0.05). **i** PASMCs were transfected with pCMV3-ENO1-GFP and treated with 100 μM etoposide for 24 h. The apoptotic markers were detected using western blotting in the cell lysate. **j** Representative images of the TUNEL assay were shown (Scale bars, 100 μm). **k** Apoptotic rates in ENO1-overexpressed PASMC under the treatment of etoposide were calculated from images in experiments depicted in **j**. *n* = 3 per group, *P = 0.04489. Data represent the mean ± SEM. Student *t* test and one-way ANOVA were used to compare two and multiple groups. Bonferroni post-tests were carried out after ANOVA

Hypertensive PASMC are more resistant to apoptosis than normal PASMC^15^. Silencing of ENO1 significantly increased the cleavage of PARP, Caspase 3, and Caspase 9 (Fig. 3a) and increased the apoptotic rate in cultured PASMC in a terminal deoxynucleotidyl transferase-mediated deoxyuridine triphosphate nick-end labeling (TUNEL) assay (Fig. 3b, c). ENOblock induced PASMC death in a dose-dependent manner (Fig. 3d), induced the cleavage of PARP, Caspase 3, and Caspase 9, and the DNA fragmentation (Fig. 3e–g). We then overexpressed ENO1 in PASMC and treated them with etoposide (an inducer of cell apoptosis). PASMC overexpressing ENO1 exhibited higher survivability under the treatment of etoposide in a dose-dependent manner (Fig. 3h). Overexpression of ENO1 also decreased etoposide-mediated cleavage of PARP, Caspase 3, and Caspase 9 (Fig. 3i) and attenuated the etoposide-induced DNA fragmentation in PASMC (Fig. 3j, k). Therefore, our data suggest that ENO1 is necessary and sufficient for the apoptotic resistance in PASMC.

### ENO1 promotes PASMC proliferation via Akt activation

**Fig. 4:**
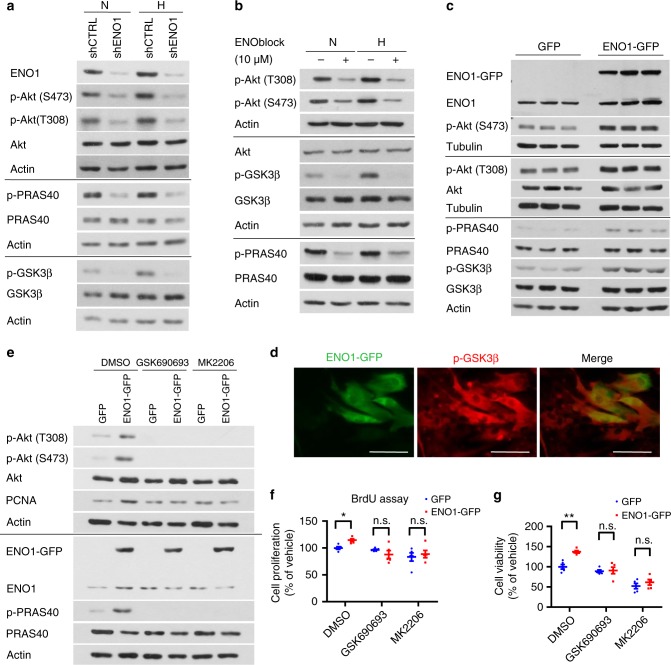
ENO1 promotes PASMC proliferation via the activation of the Akt-GSK3β pathway. **a** We treated shENO1 and shCTRL PASMC with hypoxia (1% O_2_) for 8 h, and measured the levels of p-Akt, pan-Akt, p-PRAS40, PRAS40, p-GSK3β, and GSK3β by western blotting. **b** Normal PASMCs were treated with 10 μM ENOblock and hypoxia for 8 h, and the levels of p-Akt, pan-Akt, p-PRAS40, PRAS40, p-GSK3β, and GSK3β were measured by western blotting. **c** PASMCs were transfected with pCMV3-ENO1-GFP, and afterwards we measured the level of p-Akt, pan-Akt, p-PRAS40, PRAS40, p-GSK3β, and GSK3β using western blotting**. d** PASMCs were transfected with pCMV3-ENO1-GFP and immunostained with antibody against p-GSK3β. The green fluorescence indicates the GFP-tagged ENO1 and the red fluorescence indicates p-GSK3β (Scale bars, 50 μm). **e** PASMC were transfected with pCMV3-ENO1-GFP and treated with 1 μM GSK690693 or MK2206 for 12 h. The level of p-Akt, pan-Akt, and PCNA were measured by western blotting in the cell lysate. We also measured the **f** cell proliferation (using BrdU assay) and **g** cell viability in ENO1-overexpressing PASMC after the treatment of two Akt inhibitors: GSK690693 or MK2206 (*n* = 5 per group, *P < 0.05, n.s. = non-significance). Data represent the mean ± SEM. Student *t* test and one-way ANOVA were used to compare two and multiple groups. Bonferroni post-tests were carried out after ANOVA

The Akt pathway is a key signal cascade promoting cell proliferation and survival^16^. A recent report by Tang H. et al. clearly demonstrates the dominant role of Akt1 in the development of hypoxia-induced PH^17^. We also found that hypoxia increased Akt phosphorylation at Thr308 (T308) and Ser473 (S473) and elevated phosphorylation levels of proline-rich Akt substrate of 40 kDa (PRAS40) and Glycogen synthase kinase 3β (GSK3β), two downstream targets of Akt (Fig. 4a, b). Thus, we investigated whether the Akt pathway participated in the ENO1-mediated PASMC proliferation and survival. As shown in Fig. 4a, silencing of ENO1 decreased the phosphorylation levels of Akt (at both T308 and S473), PRAS40, and GSK3β, regardless of normoxia or hypoxia conditions (Fig. 4a). Treatment of ENOblock achieved similar inhibition on phosphorylation levels of Akt, PRAS40, and GSK3β (Fig. 4b). In both shENO1 and ENOblock treatment, the total levels of Akt, PRAS40, and GSK3β remained unchanged (Fig. 4a, b). Consistently, overexpression of ENO1 significantly induced the phosphorylation levels of Akt (T308 and S473), PRAS40, and GSK3β (Fig. 4c). The GFP-positive PASMC exhibited higher level of p-GSK3β than the GFP-negative PASMC (Fig. 4d). Taken together, we show that ENO1 is sufficient and necessary for the activation of Akt/GSK3β in PASMC.

To determine whether Akt is essential for ENO1-mediated hyper-proliferation, we treated ENO1-overexpressed PASMC with two commonly used p-Akt inhibitors, GSK690693 and MK2206. GSK690693, and MK2206 completely eliminated the phosphorylation of Akt (at both T308 and S473) and PRAS40 in both control PASMC and ENO1-overexpressing PASMC (Fig. 4e). GSK690693 and MK2206 did not change levels of ENO1, total Akt, and PRAS40, however, abolished the ENO1-mediated induction of PCNA (Fig. 4e), BrdU incorporation, and viability (Fig. 4f, g). Together, these results suggest that ENO1 activates an Akt pathway to promote PASMC proliferation and survival.

### ENO1 activates Akt via AMPKα1 but independent of PEP

**Fig. 5:**
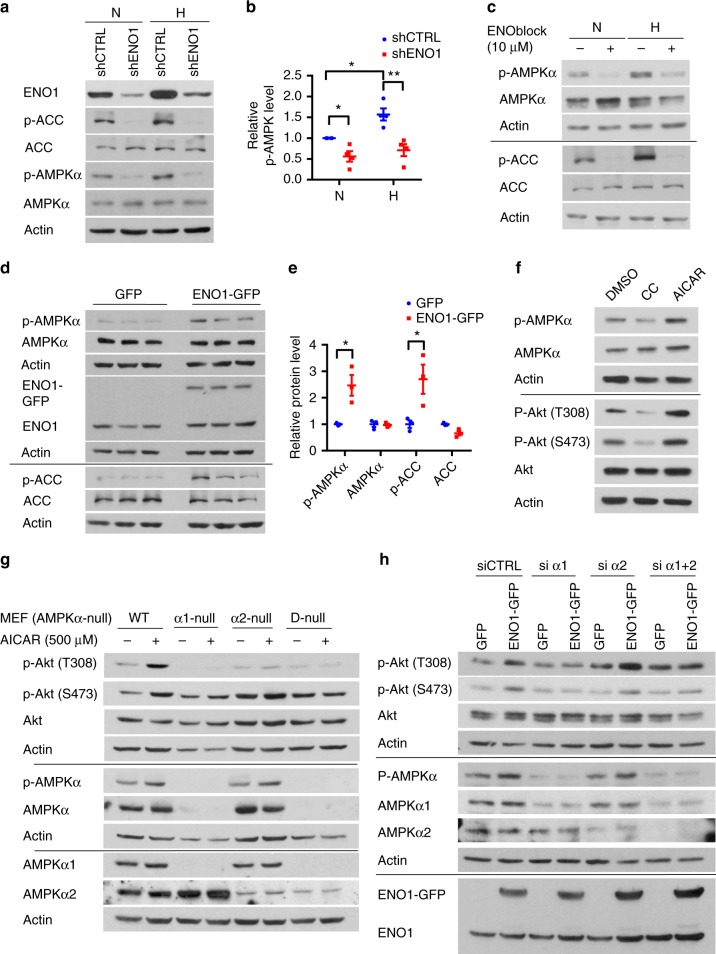
ENO1 activates Akt-GSK3β Pathway via the phosphorylation of AMPKα1. **a** We treated shENO1 and shCTRL PASMC with hypoxia (1% O_2_) for 8 h, and the levels of p-AMPKα, AMPKα, p-ACC, and ACC were measured by western blotting. **b** Normalized quantification of p-AMPKα and AMPKα in shCTRL and shENO1–PASMC under normoxic and hypoxic conditions (*n* = 4, *P < 0.05). **c** Normal PASMC were treated with 10 μM ENOblock and hypoxia for 8 h, and the levels of p-AMPKα, AMPKα, p-ACC, and ACC were measured by western blotting in cell lysates. **d**–**e** PASMC were transfected with pCMV3-ENO1-GFP, and we measured the levels of p-AMPKα, AMPKα, p-ACC, and ACC using western blotting. Actin was used as the loading control. The representative blots were shown in **d** and the quantification in **e**. *n* = 3, *P < 0.05. **f** PASMC were treated with 20 μM Compound C (CC, AMPK inhibitor) for 12 h or 500 μM AICAR (AMPK activator) for 2 h, and key proteins in the AMPK-Akt-GSK3β cascade and their phosphorylation were measured by western blotting in the cell lysates. **g** Wild-type (WT), AMPKα1-null, and AMPKα2-null MEFs were treated with 500 μM AICAR or DMSO for 2 h, and the key proteins in the AMPK-Akt cascade were measured by western blotting. **h** PASMC were overexpressed with ENO1-GFP and then transfected with siRNAs against AMPKα1 and/or α2 (si α1, si α2, or si α1 + 2), afterwards the key proteins in the AMPK-Akt cascade were measured by western blotting. Data represent the mean ± SEM. Student *t* test and one-way ANOVA were used to compare two and multiple groups. Bonferroni post-tests were carried out after ANOVA

Studies have shown that AMPK activates Akt^18,19^ and that AMPK is a key player in the hypoxia-induced PH^20^. We confirmed that hypoxia increased phosphorylation of AMPKα (T172) and acetyl-CoA carboxylase (ACC), a downstream target and standard reporter for AMPK activity (Fig. 5a–c). Silencing of ENO1 and treatment with ENOblock decreased the phosphorylation levels of AMPKα and ACC without changing total levels of AMPKα or ACC (Fig. 5a–c). Overexpression of ENO1 significantly induced the phosphorylation levels of AMPKα and ACC without alteration in total levels of AMPKα and ACC (Fig. 5d, e). These results suggest that ENO1 activates the AMPK/ACC pathway.

We treated PASMC with Dorsomorphin (Compound C, CC) or 5-Aminoimidazole-4-carboxamide ribonucleotide (AICAR) to inhibit or activate AMPK, respectively (Fig. 5f). CC inhibited the levels of p-Akt (both T308 and S473), whereas AICAR induced the phosphorylation of Akt at these sites without altering total Akt levels (Fig. 5f). To further determine which isoform of AMPKα plays a role in the activation of Akt, we treated wild-type (WT), AMPKα1-null, AMPKα2-null, and AMPKα-double null mouse embryo fibroblasts (MEFs) with AICAR. AICAR treatment significantly induced the p-Akt levels in WT MEFs, whereas the induction of p-Akt at T308 and S473 was lost in AMPKα1-null MEFs and the induction of p-Akt at T308 was lost in AMPKα2-null MEFs (Fig. 5g). This result suggests that an isoform-specific role of AMPKα in the phosphorylation of Akt: AMPKα1 targets both T308 and S473, whereas AMPKα2 targets only T308.

To determine whether ENO1 activates Akt through AMPK, PASMC were overexpressed with ENO1 and then transfected with small interfering RNAs against AMPKα1 or/and α2 (si α1, si α2, or si α1 + 2). Silencing of AMPKα1 significantly inhibited the ENO1-mediated phosphorylation of Akt at T308 and S473, whereas silencing of AMPKα2 showed little effect on Akt phosphorylation (Fig. 5h). Thus, we conclude that ENO1 activates the Akt pathway mainly via AMPKα1.

We next investigated whether PEP is responsible for ENO1-mediated activation of AMPK/Akt. We treated PASMC with PEP and confirmed that exogenous treatment of PEP was sufficient to increase intracellular PEP levels (Supplementary Fig. 6A). PEP had little effect on the levels of p-AMPK or p-Akt (Supplementary Fig. 6B), suggesting that ENO1 may activate the AMPK/Akt axis via its non-metabolic function. In our previous study, hypoxia activates AMPK rapidly and transiently: AMPK phosphorylation is maximal after 15 min exposure to hypoxia^21^. We addressed the timeline of AMPK phosphorylation, Akt activation, and ENO1 induction during hypoxia and found that hypoxia also induced the Akt-GSK3β pathway in a transient manner: phosphorylation levels of Akt and GSK3β were dramatically induced in 30 min, gradually declined afterwards, and reached a higher level compared to the control (Supplementary Fig. 7A–B). Moreover, AMPK is essential for the transient activation of Akt under hypoxia (Supplementary Fig. 7C). Interestingly, ENO1 expression was induced by hypoxia in a more delayed manner that gradually increased until 12 h (Supplementary Fig. 7A–B). Thus, ENO1 and hypoxia activate the AMPK/Akt axis through different mechanisms and dynamics.

To examine the clinical relevance of the AMPK/Akt pathway in PAH, we determined the phosphorylation levels of AMPK, ACC, Akt, and PRAS40 in PAH–PASMC. There were hyper-phosphorylation of ACC, PRAS40, and Akt (T308) in APAH but not in IPAH–PASMC compared with control PASMC (Supplementary Fig 8A–C), suggesting the activation of AMPK and Akt in APAH PASMC, correlating with upregulation of ENO1 (Fig. 1) in APAH. Interestingly, the difference of PEP levels in APAH, IPAH, and control PASMC was not statistically significant (Supplementary Fig. 8D). Thus, ENO1 may activate AMPK/Akt independently of PEP in APAH, confirming our finding above that ENO1 may activate the AMPK/Akt axis via its non-metabolic function (Figs. 4, 5, and Supplementary Fig 6).

### ENO1 regulates HIF-mediated metabolic shift via Akt

To address whether ENO1 participates in the metabolic shift in PASMC during PH and hypoxia, we mimicked the hypoxia by treating PASMC with Dimethyloxaloylglycine (DMOG), which activates HIF signaling without changing O_2_ levels. Both hypoxia and DMOG decreased the oxygen consumption rate (OCR) and increased the extracellular acidification rate (ECAR), indicating a metabolic shift toward glycolysis (Supplementary Fig. 9A–B). Silencing of ENO1 significantly enhanced the basal respiration levels (Supplementary Fig. 9C–E), reduced the basal glycolytic level (Supplementary Fig. 9B–F), and increased the basal OCR/ECAR ratio (Supplementary Fig. 9G). More importantly, silencing of ENO1 inhibited hypoxia-induced glycolysis and restored basal respiration and basal OCR/ECAR ratio (Supplementary Fig. 9E–G), suggesting that ENO1 is critical for the metabolic shift during hypoxia.

**Fig. 6:**
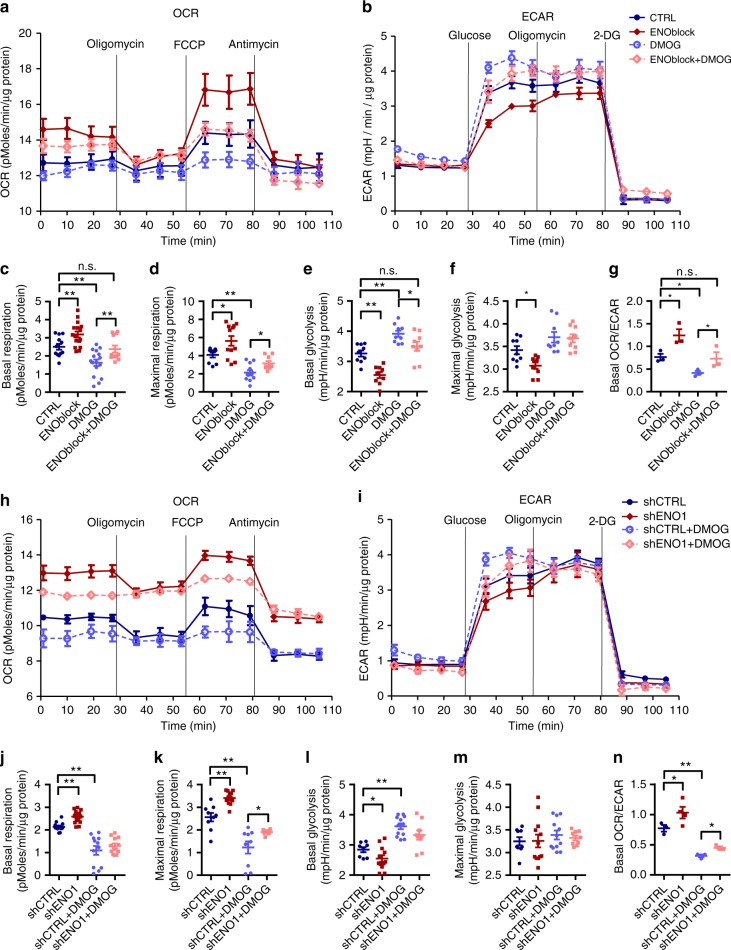
Suppression of ENO1 prevents DMOG-mediated metabolic shift in PASMC. **a** Normal PASMCs were treated with 10 μM ENOblock for 12 h before the Seahorse assay. The OCR levels were measured using the mitochondrial stress test (*n* = 3–4 per group), and **b** the ECAR levels were measured using the glycolysis stress test in ENOblock-treated PASMCs (*n* = 3 per group). DMOG was used to mimic hypoxic conditions. The basal **c** and maximal **d** respiration level, basal **e**, and maximal **f** glycolytic level, and basal OCR/ECAR **g** were calculated accordingly. **h** The OCR levels were measured using the mitochondrial stress test (*n* = 3–4 per group) and the ECAR levels were measured using the glycolysis stress test in shENO1–PASMC (*n* = 3–4 per group). The basal **j** and maximal **k** respiration level, basal **l**, and maximal **m** glycolytic level, and basal OCR/ECAR **n** were calculated. *P < 0.05, **P < 0.01, n.s. = non-significance. Data represent the mean ± SEM. Student *t* test and one-way ANOVA were used to compare two and multiple groups. Bonferroni post-tests were carried out after ANOVA

DMOG-treated PASMC exhibited lower levels of basal (Fig. 6a, c) and maximal respiration (Fig. 6d) with a greater basal glycolytic level (Fig. 6b–e) and consequently reduced basal OCR/ECAR (Fig. 6g). Treatment with ENOblock significantly enhanced the basal (Fig. 6c) and maximal respiration levels (Fig. 6d) and reduced the basal glycolytic level (Fig. 6e) in the presence or absence of DMOG. Moreover, treatment with ENOblock reversed the DMOG-induced lower OCR/ECAR to a normal level (Fig. 6g). Similarly, shENO1–PASMC displayed higher levels of basal (Fig. 6h–j) and maximal respiration (Fig. 6h, k) with a lower level of basal glycolysis (Fig. 6i–l). The basal OCR/ECAR (Fig. 6n) was also elevated in shENO1–PASMC. In the DMOG-treated PASMC, silencing of ENO1 partly reversed the DMOG-mediated lower basal (Fig. 6h–j) and maximal respiration levels (Fig. 6h–k), higher basal glycolysis (Fig. 6i–l), and lower OCR/ECAR (Fig. 6n). Taken together, these results demonstrate that ENO1 is responsible for the metabolic shift to glycolysis during hypoxia or HIF activation.

Next, we determined whether ENO1 mediates the metabolic shift via Akt activation using ENO1-overexpressed PASMC and Akt inhibitor GSK690693 (Supplementary Fig. 10). ENO1 overexpression in PASMC significantly induced the basal glycolytic level without affecting the basal respiration level, leading to a decreased basal OCR/ECAR, indicating a metabolic shift to glycolysis (Supplementary Fig. 10A–E). In control PASMC, treatment with GSK690693 significantly induced basal respiration and basal OCR/ECAR but decreased the basal glycolytic level (Supplementary Fig. 10A–E). In ENO1 overexpression PASMC, treatment with GSK690693 attenuated basal glycolysis and restored the basal OCR/ECAR (Supplementary Fig. 10C–E). Thus, ENO1 may induce the metabolic shift partly via Akt activation.

### Loss of ENO1 promotes β-oxidation and glutamine consumption

One might wonder where the energy will come from if ENO is blocked. It has been reported that there is evidence for upregulation of glycolysis and the TCA cycles in PH^22^, suggesting that the TCA cycle is utilizing alternate carbon sources, such as glutamine and/or fatty acid oxidation in PH^21,23–25^. To address whether ENO inhibition similarly shifts the TCA cycle carbon source, we measured the β-oxidation and glutamine consumption in PASMC and found that silencing of ENO1 promoted β-oxidation and increased glutamine consumption levels in PASMC (Supplementary Fig. 11A–B), suggesting that PASMC may utilize alternative energy sources after silencing of ENO1.

### Inhibition of ENO1 reverses hypoxia-induced PH in mice

**Fig. 7:**
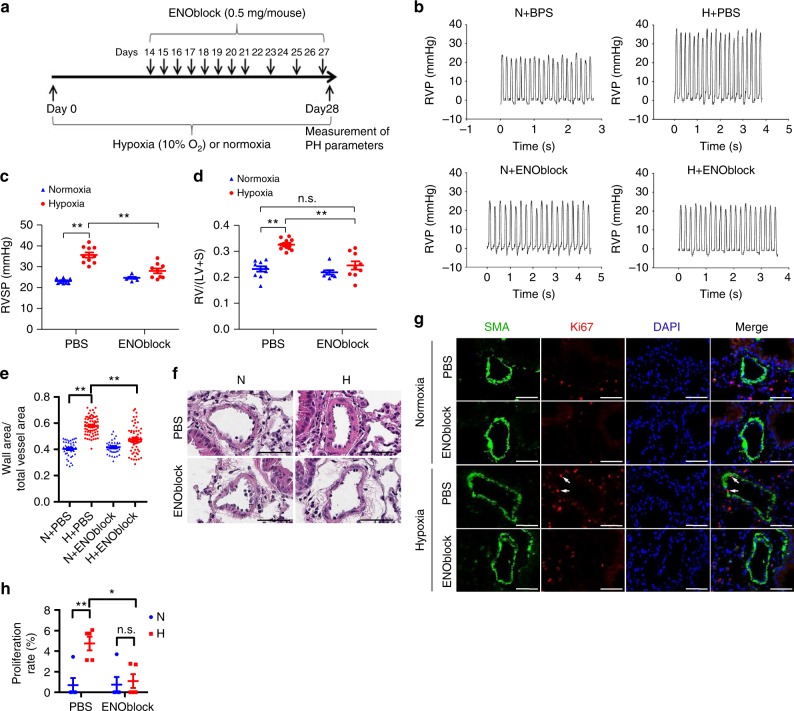
ENOblock reverses hypoxia-induced PH in mice. **a** A diagrammatic sketch depicts the hypoxia-induced PH mice model and ENOblock treatment protocol (*n* = 8–10 per group). C57BL/6 J mice were exposed to normoxia or hypoxia (10% O_2_) for 2 weeks before the ENOblock treatment. During the treatment period, the mice were continuously exposed to normoxia or hypoxia and received 0.5 mg/day/mouse ENOblock treatment via intraperitoneal injection for 2 weeks (one injection per day in the first week and one injection every other day in the second week). After the ENOblock treatment, we analyzed and calculated RVSP **c** (*n* = 8–10 per group, **P < 0.01), right ventricular hypertrophy **d** (*n* = 8–10 per group, **P < 0.01), and pulmonary artery remodeling **e** (**P < 0.01). The representative tracings of RVP are shown in **b** and the representative pulmonary artery images (HE staining) in the lung sections of experimental mice are shown in **f** (Scale bars, 50 μm). **g** The lung sections of mice from each group were co-immunostained with SMA and Ki67 antibodies and DAPI (Scale bars, 50 μm). The white arrows indicate the proliferating PASMC and **h** the proliferation rates of PASMCs were quantified in each group. Data represent the mean ± SEM. Student *t* test and one-way ANOVA were used to compare two and multiple groups. Bonferroni post-tests were carried out after ANOVA

To examine whether inhibition of ENO1 reverses PH in animal models, we utilized ENOblock to inhibit ENO1 in a commonly used hypoxia-induced mouse model of PH (Fig. 7a). We confirmed that the activity of ENO1 in hypoxic mice was significantly inhibited by ENOblock (Supplementary Fig. 12). We found that treatment of ENOblock reversed hypoxia-induced increases in the right ventricular systolic pressure (RVSP) (Fig. 7b–c), the right ventricle/(left ventricle + septum) (RV/(LV + S)) ratio (Fig. 7d), and pulmonary arterial wall thickness (Fig. 7e, f) without significant changes in the basal levels of these parameters in normoxic conditions. ENOblock dramatically inhibited hypoxia-induced hyper-proliferation (Ki67 immunostaining) in vivo (Fig. 7g, h). These results suggest that the inhibition of ENO1 with ENOblock reverses hypoxia-induced PH in vivo.

### ENOblock reverses Sugen/hypoxia-induced PH in rats

**Fig. 8:**
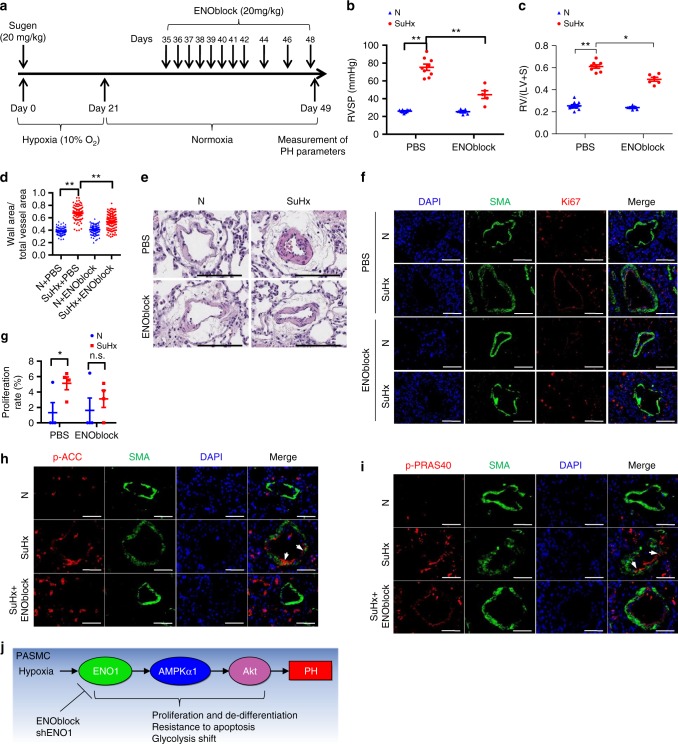
ENOblock reverses SuHx-induced PH in rats. **a** A diagrammatic sketch depicts the SuHx-induced PH rats model and ENOblock treatment protocol (*n* = 6–10 per group). Male SD rats received a dose of Sugen 5416 (20 mg/kg) subcutaneously at the first day of hypoxia exposure (10% O_2_). After 3-week chronic hypoxia exposure, the rats were placed back to room air for 2 weeks of reoxygenation before the ENOblock treatment. During the treatment period, the rats received 20 mg/kg ENOblock treatment via intraperitoneal injection for 2 weeks (one injection per day in the first week and one injection every other day in the second week). After the completion of ENOblock treatment, we measured RVP and quantified RVSP **b** (*n* = 6–10 per group, **P < 0.01), analyzed right ventricular hypertrophy **c** (*n* = 6–10 per group, **P < 0.01, *P < 0.05), and assessed pulmonary artery remodeling **d** (**P < 0.01) were analyzed and calculated accordingly. Representative HE staining images of the pulmonary arteries in the lung sections of experimental rats are shown in **e** (Scale bars, 100 μm). **f** The lung sections of experimental rats from each group were immunostained with SMA and Ki67 antibodies and DAPI to visualize the proliferating PASMC (Scale bars, 50 μm) and **g** the proliferation rates of PASMCs were quantified in each group. **h** The rat lung sections were co-immunostained with p-ACC and SMA antibodies and DAPI to visualize the AMPK activation in PASMC. The white arrows indicate the PASMC showing high p-ACC signal (Scale bars, 50 μm). **i** The rat lung sections were co-immunostained with p-PRAS40 and SMA antibodies and DAPI to visualize the Akt activation in PASMC. The white arrows indicate the PASMC showing high p-PRAS40 signal (Scale bars, 50 μm). **j** A schematic diagram depicts the role of the ENO1-AMPK-Akt axis in PH. ENO1 activates the AMPKα-Akt signaling cascade, therefore promotes PASMC proliferation, de-differentiation, apoptotic resistance, and metabolic shift, leading to PH. Inhibition of ENO1 represents a viable therapeutic strategy for PAH. Data represent the mean ± SEM. Student *t* test and one-way ANOVA were used to compare two and multiple groups. Bonferroni post-tests were carried out after ANOVA

Combination of the Sugen 5416 injection and hypoxia exposure (SuHx) induces a much more profound PH phenotype in rats. To further test the therapeutic effects of ENOblock in PH, we also adopted the ENOblock treatment in the SuHx-induced rat model of PH (Fig. 8a). ENOblock did not change the basal levels of PH parameters in normoxic rats (Fig. 8b–e). However, ENOblock attenuated the SuHx-induced development of PH as assessed by the inhibition of RVSP (Fig. 8b), RV/(LV + S) ratio (Fig. 8c) and pulmonary arterial wall thickness (Fig. 8d–e). Ki67 and SMA co-immunostaining showed that ENOblock treatment inhibited hypoxia-induced PASMC hyper-proliferation in vivo (Fig. 8f, g).

To address whether ENOblock inhibits PH via inhibition on AMPK/Akt, we tested the effects of ENOblock treatment on the AMPK/Akt activation in SuHx-induced PH rats. ENOblock treatment significantly inhibited the p-ACC and p-PRAS40 in SuHx-induced PH rat lungs and PASMC (Fig. 8h, i), suggesting that ENOblock may inhibit PH in vivo via its inhibition on the ENO/AMPK/Akt pathway (Fig. 8j).

We then addressed whether AMPK/Akt contributes to the ENO1-mediated PASMC phenotype changes. We found that APAH PASMC also expressed decreased levels of myocardin and MHC (Supplementary Fig. 13), confirming the de-differentiated phenotype of APAH PASMC. ENOblock restored myocardin and MHC expression in SuHx rats (Supplementary Fig. 14). Furthermore, we silenced ENO1 in three representative lines of PASMC from each group of control, IPAH, and APAH (Supplementary Fig. 15). Silencing of ENO1 decreased PASMC proliferation, induced myocardin expression, cell death and Caspase 3 cleavage, and correlated with the reduction in p-ACC and p-PRAS40, regardless the source of these cells (Supplementary Fig. 15A–I). Thus, inhibition of ENO1 is sufficient to restore normal PASMC phenotype in hypertensive cells. Taken together, our data suggest that ENO1 inhibition may limit PH by preventing AMPK/Akt activation (Fig. 8j).

## Discussion

There are reports showing the presence of ENO1 antibodies in the serum of systemic sclerosis patients with PAH^26^ and that ENO1 antibodies in serum induce vascular smooth muscle cell contraction^27^. In this study, we investigated the contribution of ENO1 in the pathogenesis of PAH using molecular and biological approaches, human samples, and animal models. We have discovered that ENO1 expression is upregulated in PASMC isolated from patients with APAH and in experimental PH lungs (Fig. 1, Supplementary Fig. 1–2). We have identified a conserved ENO1/AMPKα1/Akt signaling axis in control and diseased PASMC that regulates PASMC proliferation, de-differentiation, resistance to apoptosis (Figs. 2–5, Supplementary Fig. 13–15) and the hypoxia-induced metabolic shift (Fig. 6, Supplementary Fig. 9–10). Importantly, we have found that inhibition of ENO1 is sufficient to reverse existing PH in two animal models (Figs. 7, 8, supplementary Fig. 14). Our study provides the evidence for the regulatory role of ENO1 in the pathogenesis of PAH (Fig. 8j).

The pathogenesis of PH is very complex and differs among different types, involving multiple modulating genes and environmental factors^16^. Elevated ENO1 levels are found in APAH PASMC but not in pulmonary artery endothelial cells (PAEC) and fibroblasts or in IPAH (Fig 1a–d), confirming a differentiated etiology between IPAH and APAH. These results indicate that, the ENO1 level is specifically elevated in PASMC of APAH patients. This notion needs further validation with a large number of APAH samples. The selective ENO1 staining in the media of the pulmonary artery of APAH and IPAH patients (Fig. 1d) may also indicate a specific ENO1 upregulation in subpopulations of PASMC due PASMC heterogeneity. In experimental PH models, we also noticed a model-specific regulation of ENO1: ENO1 is upregulated in hypoxia-induced PH mice and SuHx-induced PH rats, (Fig. 1), but not in MCT-induced PH rats (Supplementary Fig. 1A–D). These results suggest that ENO1 upregulation is PH type-specific.

Despite the different etiologies, PH PASMC all share hyperproliferative feature in early stage and apoptosis resistance phenotypes, implying that therapies targeting these key pathways may be effective in multiple forms of PH. Although we showed differences in ENO1 levels, AMPK and Akt activation between APAH–PASMC and IPAH–PASMC (Supplementary Fig. 8), there is no significant difference in PASMC proliferation between IPAH and APAH (Supplementary Fig. 16) and the ENO1-AMPK-Akt pathway is conserved in all tested control and diseased PASMC (Supplementary Fig. 15), suggesting that they may be common targets for PH therapies. Indeed, ENOblock reverses PH in two experimental models that are commonly used to study PH in preclinical studies (Figs. 7, 8). However, it is worth noting that ENO1 controls PASMC proliferation, de-differentiation, apoptosis resistance, metabolic switch whether ENO1 is upregulated or not (Figs. 2–6), suggesting that the effects of ENO1 on these features are intrinsic to basal levels of ENO1, similar to ENO-silenced pancreatic carcinoma^28^. Thus, therapies with ENO1 inhibitors may have side effects that could impact physiologic and adaptive cell proliferation.

Although PASMC are well-differentiated cells, they demonstrate a certain degree of plasticity^15^. Our study shows that ENO1 is necessary and sufficient for PASMC proliferation, de-differentiation, and resistance to apoptosis (Figs. 2, 3 Supplementary Fig. 14–15). ENO1-mediated de-differentiation is partial, as it only affects the expression of certain contractile proteins. This suggests that multiple signaling pathways contribute to the regulation of contractile protein expression^14^. It is also possible that ENO1 regulates PASMC proliferation and de-differentiation in certain subpopulations of PASMC owing to its heterogeneity. Nonetheless, ENO1 is a key regulator of PASMC plasticity in the development of PH.

Several studies reported the regulatory roles of the Akt pathway in PAH. Akt1-knockout mice are found more protected against the development of hypoxia-induced PH^17^. Akt downstream targets such as mTOR and Forkhead box O are implicated in PH^29–31^. Consistently, we have shown that the PASMC of SuHx-PH rats and APAH patients exhibits higher p-PRAS40 levels indicating the activation of Akt pathway (Fig. 8h, Supplementary Fig. 8A–C). Our study reveals that elevated ENO1 promotes PASMC proliferation and metabolic shift via the activation of the Akt pathway (Fig. 4, Supplementary Fig. 10) and that ENOblock treatment significantly inhibited AMPK and Akt activations in vivo (Fig. 8h, i). These results clearly demonstrate the participation of Akt in ENO1-mediated PH.

The role of AMPK in PH appears to be controversial: some studies show that AMPK activators metformin and AICAR are protective against experimental PH^32,33^, whereas other studies demonstrate that AMPK activation induces vasoconstriction and that inhibition of AMPK by Compound C prevents and reverses hypoxia-induced PH^20,34–36^. Our data clearly show that the p-ACC level is elevated in the PASMC isolated from patients with APAH (Supplementary Fig. 8A–C) and in the SuHx-PH rats (Fig. 8h) and that AMPKα1 is the main mediator for ENO1-activated Akt phosphorylation in PASMC (Fig. 5). Interestingly, hypoxia and ENO1 activate AMPK-Akt-GSK3β axis via different time frame (Supplementary Fig. 7). Given the fact that PAH PASMC contains constitutive high AMPK phosphorylation^20^, we reason that ENO1 is responsible for maintaining activation of the AMPK-Akt-GSK3β axis during PAH.

During PH, there is a metabolic shift to glycolysis, similar to the Warburg effect in cancer cells^3,37^. The shift to glycolysis increases the glucose consumption to provide a carbon source for cell proliferation^38^ and engages cells in local metabolite-based paracrine and autocrine signaling for growth^39^. Therefore, the inhibition of glycolysis pathway has become a compelling rationale for therapeutic modalities^40^. In cancer and PAH, such glycolytic enzyme targets include Hexokinase, glucose 6 phosphate dehydrogenase (G6PD), phosphofructokinase 2/fructose bisphosphatase 3 (PFK2/PFKFP3), pyruvate kinase 2 (PKM2), lactate dehydrogenase, and pyruvate dehydrogenase kinase^41^. 2-Deoxyglucose (2-DG) blocks the glucose metabolism by inhibiting HK2^42^, decreases free NADH and inhibits fibroblast and vascular smooth muscle cells proliferation^43,44^. Dehydroepiandrosterone inhibits G6PD and alleviates PH phenotype^45^. PFK2/PFKFP3 inhibitor 3-(3-pyridinyl)-1-(4-pyridinyl)-2-propen-1-one abolishes angiogenesis and TGF-β1–induced lung fibrosis^46^. In both PAEC and fibroblasts, a microRNA-124/ Polypyrimidine Tract Binding Protein 1/PKM2 axis is responsible for hyper-proliferation and metabolic shift during PH and can be targeted for PH therapy^47,48^. Ranolazine and Trimetazidine can reduce HK1 and LDHA to protect animals from PH^49^. Most convincingly, dichloroacetate inhibits PDK1, activates pyruvate dehydrogenase, and reverses the glycolytic shift and the resistance to apoptosis, leading to the inhibition of PH in experimental models^50,51^ and in genetically susceptible PAH patients^52^. These enzymes are either irreversible (HK, PKM) or rate limiting (G6PD, PFK), or allosteric (PFK), making them less druggable. We show that inhibition of ENO1 can reverse the experimental PH in animals, expanding the armamentarium for PAH therapy. The advantage of targeting ENO1 may be twofold: (1) the glycolytic step catalyzed by ENO1 is reversible, providing the flexibility of drug dosing and frequency; (2) there are three isoforms of ENO1, whereas only ENO1 is dysregulated in PH, leading to the target specificity and less toxicity.

Although the inhibition of metabolic activity by ENOblock is consistent throughout the literature, the exact mechanism of action of ENOblock in vivo may be complex and not fully understood and even controversial^53^. Some studies support the direct inhibition of ENO by ENOblock^54,55^, whereas other researchers argue that such effects may be caused by mechanisms other than direct inhibition of ENO activity^56^. We show that ENOblock can inhibit ENO activity both in vitro and in vivo (supplementary Fig. 4, 9) and displays a robust therapeutic effect on both hypoxia-induced PH in mice and SuHx-induced PH in rats (Fig 7, 8). In cell-based studies, although both ENO1 silencing and ENOblock treatment in general inhibit PASMC proliferation and induce contractile protein expression, they show different effects on selected SMC contractile protein levels (Fig. 2e, j), possibly owing to non-specific or off-target effects of ENOblock. Thus, genetic approaches that specifically target ENO1 are more helpful for a mechanistic understanding of the role of ENO1 in PH. Owing to the developmentally lethal phenotype of *Eno1*-knockout (KO) mice and the unavailability of the *Eno1*-floxed mice, it is currently impossible to test the participation of ENO1 in PH with *Eno1*-KO mice.

As a glycolytic enzyme, ENO1 may directly participate in the hypoxia-mediated metabolic shift in PASMC. We have found that inhibition or silencing of ENO1 successfully restores the mitochondrial respiration from glycolysis (Fig. 6, Supplementary Fig. 9–10). This is consistent with the metabolic phenotype of ENO-silenced pancreatic carcinoma^28^, highlighting certain similarities between PAH and cancer^3,37^. When ENO1 is inhibited or silenced, PASMC maintain basal respiration (Fig. 6, Supplementary Fig 9–10). Previous reports show the feed to TCA and respiration can be from alternative sources, such as glutamine and/or fatty acid oxidation^21,23–25^. Consistently, there is a compensatory increase in β-oxidation and glutamine consumption after silencing of ENO1 (Supplementary Fig. 11), which restores the acetyl-CoA bulk and thus increases anaplerosis via the TCA cycle. It is worth mentioning that the Akt pathway is also a critical regulator of glycolysis. Akt has been shown to induce glucose uptake by regulating the localization of the glucose transporter 1 to the plasma membrane^57^. In addition, Akt can activate the glycolytic rate-controlling enzyme phosphofructokinase 1 by directly phosphorylating PFK2^58,59^. Our results show that Akt inhibition partially blocks the exogenous ENO1-induced metabolic shift in PASMC (Supplementary Fig. 10). Thus, ENO1 may partially regulate the metabolic shift by the activation of Akt. However, activation of AMPK/Akt appears to be independent of PEP, the ENO1 enzymatic product (Supplementary Fig. 6, 8D), suggesting a non-metabolic function of ENO1 in AMPK/Akt activation, PASMC proliferation, de-differentiation, and resistance to apoptosis. This is consistent with other reports on the non-metabolic function of ENO1 as a plasminogen receptor^11^ or as a nuclear protein MBP-1^60^.

Although we have shown the participation of ENO1 in PAH, particularly hypoxic pulmonary hypertension (HPH), our study has limitations. One limitation is that we only studied PASMC, whereas other vascular cell types, particularly PAEC, fibroblasts, monocytes/macrophages are also critical for the pathogenesis of PAH^61^. In the lung sections, ENO1 was present in these cell types (Fig. 1d, Supplementary Fig 2). More importantly, in hypoxia-PH mice and SuHx-PH rats, ENO1 is also elevated in many other cell types, including PAEC and fibroblasts (Figs. 7g and 8f, Supplementary Fig. 2). Failure to resolve inflammation and altered immune processes have been long considered an underpinning mechanism for the development of PAH^62^. Recent data show that ENO1 promotes monocytes recruitment in inflamed lungs^63^ and that ENO1 in monocytes and macrophages induces robust synovial inflammation in rheumatoid arthritis^64^. Thus, we cannot exclude the possibility that ENO1 may be involved in phenotypical changes of other cell types such as PAEC, fibroblasts, monocytes/macrophages during PH.

Another limitation is that at this stage we do not know what causes the preferential upregulation of ENO1 in APAH and HPH as compared with IPAH or MCT-induced PH. Until now, HIF is the best-known inducer of ENO1, therefore, it is not surprising that ENO1 was upregulated in hypoxia and Sugen/Hypoxia-induced rodent models of PH (Fig. 1). ENO1 can regulate the metabolic shift in PASMC exposed to hypoxia or treated with DMOG, which increases HIF-activity without changing in O_2_ levels (Fig. 6, Supplementary Fig. 9–10). Thus, ENO1 upregulation in certain forms of PH is likely a HIF-dependent phenomenon. APAH samples include patients with collagen vascular disease/connective tissue disease and congenital systemic-to-pulmonary shunts (Supplementary Table 1). There is a varying inflammation between MCT-induced PH and HPH. Thus, abnormality in growth factors, cytokines, and inflammatory factors during APAH or HPH may also be responsible for induction of ENO1 in APAH. How ENO1 is upregulated in APAH warrants further study.

In summary, our results demonstrate that there is a dysregulation of ENO1 in PAH and hypoxia related PH and that inhibition of ENO1 is sufficient and efficient to protect animals from PH. Mechanistically, we have provided the axis of ENO1-AMPK-Akt that controls the cancer-like PASMC phenotypes characterized by increased cell proliferation, de-differentiation, apoptosis resistance, and metabolic shift to glycolysis. Thus, the ENO1-AMPK-Akt pathway regulates the pathogenic metabolic reprogramming observed in PASMC during PH.

## Methods

### Cell culture and hypoxic exposure

Primary human PASMC (CC-258) were obtained from Lonza (Walkersville, MD). We obtained human PASMC samples from normal donors, patients with IPAH, and APAH from the PHBI^14^. APAH samples include patients with collagen vascular disease/connective tissue disease and congenital systemic-to-pulmonary shunts. Supplementary Table 1 shows the demographics, hemodynamic parameters, and 6MWD for PAH patients obtained from PHBI. We have complied with all relevant ethical regulations regarding these cells and use of them was approved by the University of Illinois at Chicago Institutional Review Board. Informed consent was obtained from all human participants. Cells were cultured at 37 °C in SmGM-2 media (Lonza, Walkersville, MD) that contain 5% fetal bovine serum (FBS), growth factors, and 1% penicillin–streptomycin. Because of the plasticity of PASMC, culture condition and source of PASMC may complicate the experimental result or data interpretation. To overcome this limitation, we have used lower passages (passage 5–7) PASMC, which will give us sufficient cells for the experiments while enable us to maintain the PASMC phenotype. For experiments with ENO1 silencing or overexpression, we used passage-matched PASMC between ENO1 intervention group and control group. As we utilized human PASMC purchased from Lonza and PASMC provided by PHBI, we examined whether the source of PASMC makes difference. To address that, we compared the cell proliferation (a key parameter in our studies) between human PASMC from Lonza and normal human PASMC from PHBI by two different methods. As shown in Supplementary Fig. 17A–B, the cell proliferation between PASMC from Lonza and PASMC from PHBI is comparable. Wild-type (WT), AMPK α1-null, AMPK α2-null, and AMPK α-double null MEFs were cultured at 37 °C in Dulbecco's Modified Eagle Medium (DMEM) and supplemented with 5% FBS. Hypoxia (1% O_2_) was achieved using an INVIVO2 300 hypoxia chamber (Ruskinn Technology Limited, Bridgend, UK) with an O_2_ sensor.

### Cell proliferation, cell viability, cytotoxicity, and apoptosis

Cell proliferation was determined by the BrdU incorporation assay (EMD Millipore Corporation, Germany). Cell viability was measured using the Cell Titer 96 AQueous One Solution Cell Proliferation Assay (Promega Corporation, Madison, WI). Cell death levels were measured with the Cytotoxicity lactate dehydrogenase Detection Kit (Roche, Mannheim, Germany). A GloMax®-96 Microplate illuminometer (Promega, Madison, WI) was used to read the 96-well plates. Cell apoptosis rates were detected by the TUNEL assay (Abcam, Cambridge, UK).

### Silencing and overexpression of ENO1 in PASMC

To stably silence *ENO1*, PASMC were infected by lentivirus containing shRNA targeting *ENO1* (Santa Cruz Biotechnology, Dallas, TX). Two days after infection, we cultured these cells in a medium containing 1.5 mg/ml puromycin (Sigma-Aldrich, St. Louis, MO) for 2 days to remove uninfected cells. To transiently overexpress ENO1, PASMC were transfected with pCMV3-ENO1-GFP (Sino biological, China) using the Nucleofector Electroporation System (Lonza, Basel, Switzerland). A blank pCMV3-GFP plasmid was also transfected as a negative control.

### Measurement of ENO activity and PEP levels

ENO activity in mouse whole lung tissue or PASMC was measured using the Enolase Activity Kit (Biovision, Milpitas, CA). The level of PEP was measured using the PEP Colorimetric/Fluorometric Assay Kit (Abnova, Taipei, Taiwan) according to the manufacturer’s protocol.

### Western blot analysis

Protein samples were subjected to SDS-polyacrylamide gel electrophoresis. After electrophoresis, the separated proteins were transferred onto nitrocellulose membranes. The membranes were blocked in 5% skim milk for 1 h and subsequently incubated overnight with diluted primary antibodies at 4 °C. After rinsing three times with tris-buffered saline (TBS) with tween 20, the membranes were incubated with secondary antibodies for 1 h at room temperature. After rinsing, the bands were detected using the SuperSignal West Femto Substrate (Thermo Fisher, Waltham, MA), and the blot was exposed to X-ray film. The films were scanned, and the gray density of protein bands was determined using ImageJ. All the uncropped films are provided in Supplementary Fig. 18–36. The following primary antibodies were used in this study: α-Tubulin (Cat#T5168, 1:2000, Sigma-Aldrich, St. Louis, MO), β-actin (Cat#A2228, 1:2000, Sigma-Aldrich, St. Louis, MO), α-SMA (Cat#A5228, 1:1000, Sigma-Aldrich, St. Louis, MO), Calponin (Cat#C2687, 1:1000, Sigma-Aldrich, St. Louis, MO), Myocardin (Cat#MAB4028, 1:1000, R&D Systems, Minneapolis, MN), SM22α (Cat#ab10135, 1:1000, Abcam, Cambridge, MA), PCNA (Cat#10205–2-AP, 1:1000, Proteintech Group, Chicago, IL), ENO1 (Cat#3810, 1:1000, Cell Signaling Technology, Danvers, MA), PARP (Cat#9542, 1:1000, Cell Signaling Technology, Danvers, MA), Cleaved-PARP (Cat#5625, 1:1000, Cell Signaling Technology, Danvers, MA), Caspase 3 (Cat#9665, 1:1000, Cell Signaling Technology, Danvers, MA), Cleaved-Caspase 3 (Cat#9664, 1:1000, Cell Signaling Technology, Danvers, MA), Caspase 9 (Cat#9508, 1:1000, Cell Signaling Technology, Danvers, MA), Cleaved-Caspase 9 (Cat#7237, 1:1000, Cell Signaling Technology, Danvers, MA), p-PRAS40 (Cat#13175, 1:1000, Cell Signaling Technology, Danvers, MA), PRAS40 (Cat#2691, 1:1000, Cell Signaling Technology, Danvers, MA), p-Akt (T308) (Cat#13038, 1:1000, Cell Signaling Technology, Danvers, MA), p-Akt (S473) (Cat#4060, 1:1000, Cell Signaling Technology, Danvers, MA), Akt (Cat#4691, 1:1000, Cell Signaling Technology, Danvers, MA), p-GSK3β (Cat#5558, 1:1000, Cell Signaling Technology, Danvers, MA), GSK3β (Cat#12456, 1:1000, Cell Signaling Technology, Danvers, MA), p-ACC (Cat#11818, 1:1000, Cell Signaling Technology, Danvers, MA), ACC (Cat#3676, 1:1000, Cell Signaling Technology, Danvers, MA), p-AMPKα (T172) (Cat#2535, 1:1000, Cell Signaling Technology, Danvers, MA), AMPKα (Cat#5831, 1:1000, Cell Signaling Technology, Danvers, MA), AMPKα1 (Cat#07-350, 1:1000, Upstate Biotechnology, Fairport, NY), AMPKα2 (Cat#07-363, 1:1000, Upstate Biotechnology, Fairport, NY), MHC (Cat#sc-376157, 1:1000, Santa Cruz Biotechnology, Santa Cruz, CA), ENO2 (Cat#sc-71047, 1:1000, Santa Cruz Biotechnology, Santa Cruz, CA), ENO3 (Cat#sc-100811, 1:1000, Santa Cruz Biotechnology, Santa Cruz, CA), and HIF1α (Cat#610958, 1:1000, BD Biosciences, San Jose, CA). The anti-mouse (Cat#172–1011, 1:10000, Bio-Rad, Hercules, CA), anti-rabbit (Cat#172–1034, 1:20000, Bio-Rad, Hercules, CA), and anti-goat (Cat#172–1019, 1:10000, Bio-Rad, Hercules, CA) IgG-HRP conjugates were use as secondary antibodies.

### RNA isolation and real-time polymerase chain reaction

Total RNA from cells or tissues was isolated using the RNeasy Plus Mini Kit (Qiagen, Valencia, CA). Two micrograms of purified RNA were reverse transcribed to single-stranded cDNA using the Taqman RNA reverse transcription kit (Applied Biosystems, Foster City, CA). Real-time polymerase chain reaction (PCR) was performed on a StepOnePlus Real-Time PCR System (Applied Biosystems, Foster City, CA). The information of the primers used in the Real-time PCR were listed in Supplementary Table 2. The relative quantifications were performed using the 2^−Δ(ΔCt)^ method, where the relative quantification or fold-change is equal to 2^−((Mean ΔCt Target) − (Mean ΔCt Calibrator))^.

### Immunofluorescence (IF) and immunohistochemistry (IHC)

Lung tissue sections from normal donors and PAH patients were provided by Drs. Suzy A, A. Comhair and Serpil C. Erzurum at Department of Pathobiology, Respiratory Institute, Cleveland Clinic. Use of these tissue sections was approved by the University of Illinois at Chicago Institutional Review Board. The tissue sections were baked at 85 °C for 15 min and subsequently de-paraffinized and rehydrated according to standard protocols. After antigen retrieval in 0.01 M citrate buffer (pH 6.0), the tissue sections were incubated with 1% Triton X-100 for 15 min and incubated with 5% bovine serum albumin (in TBS, pH = 7.4) for 30 min, followed by overnight incubation with the primary antibodies at 4 °C. After a wash with TBS, the sections were incubated with the secondary antibodies at room temperature for 1 h. After washed three times with TBS, the sections were mounted using the 496-diamidino-2-phenylindole mounting medium. The fluorescence was recorded with a ZEISS Z1 AxioObserver fluorescence microscope (Carl Zeiss, Oberkochen, Germany). The proliferation rates of PASMC were quantified as the ratio of Ki67-positive/SMA position over SMA-positive cells. For the IHC staining, sections were incubated with horseradish peroxidase-labeled secondary antibody for 30 min at room temperature. The peroxidase reaction was developed using 3, 3-diaminobenzidine (DAB) chromogen solution in DAB buffer (Dako, Glostrup, Denmark). Sections were visualized with DAB and counterstained with hematoxylin, mounted in neutral gum, and analyzed using a bright field microscope. The following primary antibodies were used in the IF and IHC assays: α-SMA (Cat#A5228, 1:200, Sigma-Aldrich, St. Louis, MO), Myocardin (Cat#MAB4028, 1:100, R&D Systems, Minneapolis, MN), MHC (Cat#sc-376157, 1:1000, Santa Cruz Biotechnology, Santa Cruz, CA), ENO1 (Cat #3810, 1:100), p-ACC (Cat #11818, 1:50), p-PRAS40 (Cat #13175, 1:50) (Cell Signaling Technology, Danvers, MA).

### Oxygen consumption and extracellular acidification

OCR and ECAR were measured using Seahorse XF24 Cellular Flux Analyzer (Agilent, Santa Clara, CA) in two different test conditions: the mitochondrial stress test and the glycolysis stress test. Thirty thousand PASMC per well were seeded in Seahorse XF24 cell culture microplates and cultured in a 37 °C incubator containing 5% CO_2_ for 12 h. After washing with assay medium (non-buffered XF Base Medium Minimum DMEM) PASMC were kept in a CO_2_‐free incubator at 37 °C for 1 h before the assay. DMOG (100 μM) was added to mimic the hypoxic condition. During the mitochondrial stress test, selective inhibitors were injected during the measurements to achieve the final concentrations of oligomycin (1 μM), Carbonyl cyanide-4-(trifluoromethoxy) phenylhydrazone (5 μM), and antimycin (1 μM). For the glycolysis stress test, three injections were performed as follows: glucose (20 mM), oligomycin (4.5 μM), and 2-DG (100 mM). For ENOblock-treated wells, ENOblock (10 μM) was added after PASMC were attached to the plate and incubated for 12 h and during the test. After the OCR or ECAR measurement, cells from each well were lysed and protein concentrations were determined. The OCR and ECAR values were further normalized to the protein amount in each well. The respiration/glycolysis parameters were calculated as follows: basal respiration level = (basal OCR)–(non-mitochondrial respiration), maximal respiration level = (maximal OCR capacity)–(non-mitochondrial respiration), basal glycolytic level = (basal ECAR)–(non-glycolytic acidification), maximal glycolytic level = (maximal ECAR capacity)–(non-glycolytic acidification), basal OCR/ECAR = [(basal OCR)–(non-mitochondrial respiration)]/ = [(basal ECAR)–(non-glycolytic acidification)].

### β-oxidation and glutamine consumption

The β-oxidation levels in PASMC were measure using the Fatty Acid Oxidation Assay Kit (Abcam, Cambridge, United Kingdom) and the OCR level were measured using the Seahorse system. Glutamine consumption level in cell medium were measured by using the Glutamine-Glo Assay Kit (Promega, Madison, WI) as an indicator of the glutaminolysis levels.

### HPH models and ENOblock treatment

A hypoxia-induced mouse PH model and a SuHx-induced rat PH model were used in our study and the experimental protocols were approved by our Institutional Animal Care and Use Committee. We have complied with all relevant ethical regulations. All animals were purchased from Jackson Laboratory (Bar Harbor, ME) and were handled according to the National Institutes of Health (NIH) guidelines. For the hypoxia-induced mice PH model, 8-week-old C57BL/6 J mice were exposed to room air (normoxia) or 10% oxygen (hypoxia) in a BioSpherix A-chamber (BioSpherix, Lacona, NY), and the oxygen concentration was monitored by a Proox Model P110 oxygen controller (BioSpherix, New York, USA). Equal number of male and female mice were exposed to normoxia or hypoxia for 2 weeks before the ENOblock treatment. During the treatment period, the mice were continuously exposed to normoxia or hypoxia and received 0.5 mg per day per mouse ENOblock treatment via intraperitoneal injection for 2 weeks (one injection per day in the first week and one injection every other day in the second week). There were 8–10 mice in each experimental group. Male Sprague-Dawley rats (190–200 g, 6-week old) were used for the SuHx-induced PH studies and each group included 6–10 rats. A dose of Sugen 5416 (20 mg kg^−1^) was given subcutaneously via subcutaneous injection at the first day of hypoxia exposure (10% O_2_). After 3-week chronic hypoxia exposure, the rats were placed back to room air for 2 weeks of reoxygenation. Afterwards, ENOblock treatment (20 mg kg^−1^) via intraperitoneal injection was started and continued for 2 weeks (one injection per day in the first week and one injection every other day in the second week).

After the treatment, RVSP was measured by right heart catheterization using a Millar pressure transducer catheter. A weight ratio of the RV divided by the sum of LV and S (RV/[LV + S]) was determined as an index for right ventricular hypertrophy (RVH). Pulmonary artery remodeling was assessed using Aperio ImageScope software (version 11) after lung tissue sections were stained with hematoxylin and eosin. A minimum of 10 muscular arteries (with diameters ranging from 50 to 100 µm for the mice, and 60 to 120 µm for the rats) per lung section were outlined and included in the analysis. Vessel remodeling index was calculated as (external vessel area–internal vessel area)/external vessel area^65^. The animal studies were randomized (block randomization) and blinded.

### Statistical analysis

For animal studies, we estimated the sample size with an online power analysis tool (http://www.datavis.ca/online/power/index.html), adopting a moderate to large difference (effect size 0.75). Statistical analysis of experimental data was performed using GraphPad Prism 6.0 (GraphPad Software, La Jolla, CA). Results are expressed as mean ± SEM from at least three experiments. Student *t* test and analysis of variance were used to compare two and multiple groups, respectively. Bonferroni post-tests were carried out after analysis of variance. Significant differences are indicated by * (*P* *<* 0.05), and very significant differences are indicated by ** (*P* < 0.01).

## Electronic supplementary material

Supplementary information

## Data Availability

The authors declare that all relevant data supporting the findings of this study are available within the paper and its supplementary information files. All data are available from the authors upon reasonable request.
