# Supplementary material for: Alpha-enolase regulates the malignant phenotype of pulmonary artery smooth muscle cells via the AMPK-Akt pathway
Source: Nat Commun. 2018 Sep 21;9:3850. doi: 10.1038/s41467-018-06376-x (PMC6155017; doi:10.1038/s41467-018-06376-x)
Supplement: Supplementary file 1 — Supplementary information [file 41467_2018_6376_MOESM1_ESM.pdf]

Alpha-Enolase Regulates the Malignant Phenotype of Pulmonary Artery Smooth Muscle Cells via the AMPK-Akt pathway

Dai et al.

Supplementary information

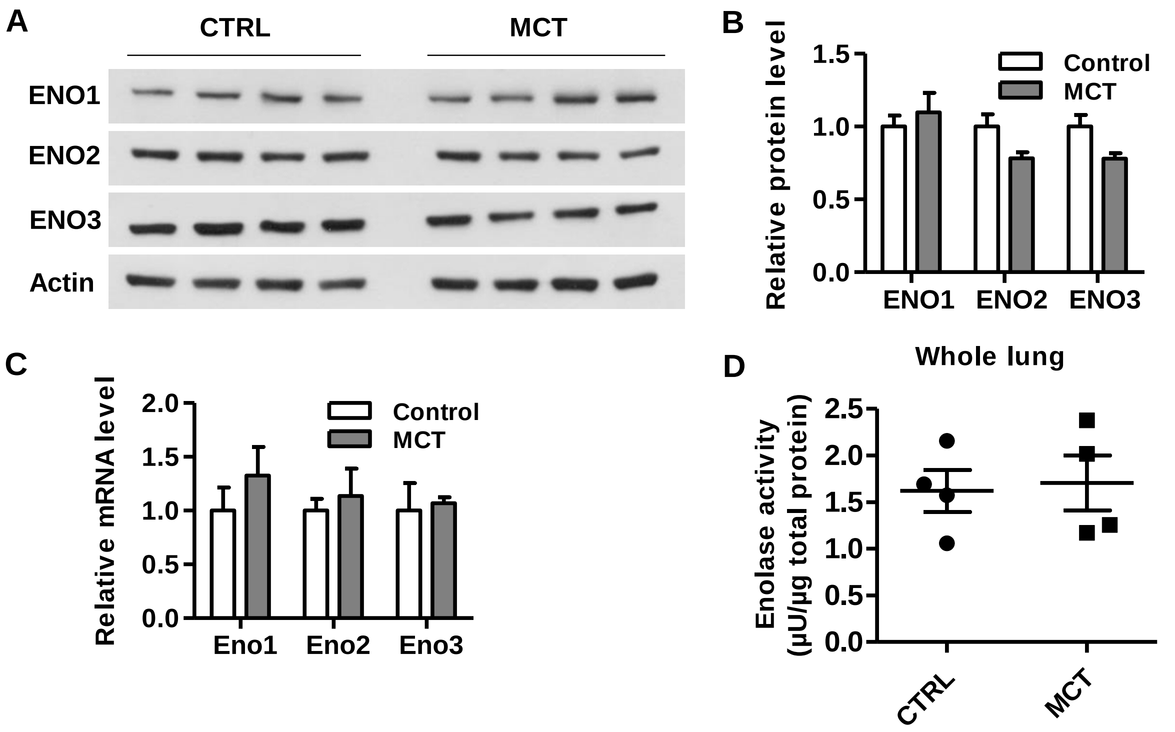

Supplementary Figure 1. Enolase expression or activity are unchanged in MCT-rat PH model.

(A) Whole lung tissues were isolated from MCT-induced PH rats and Western blotting was used to measure the level of enolase. (B) Normalized quantification of protein and (C) the mRNA level demonstrate the expression level of ENO1, ENO2, and ENO3 in whole lung from MCT treated rat PH model. (D) the enolase activity in whole lung from MCT treated rat PH model (n=4). Data represent the mean ± SEM. Student t test and one-way ANOVA were used to compare two and multiple groups. Bonferroni posttests were carried out after ANOVA.

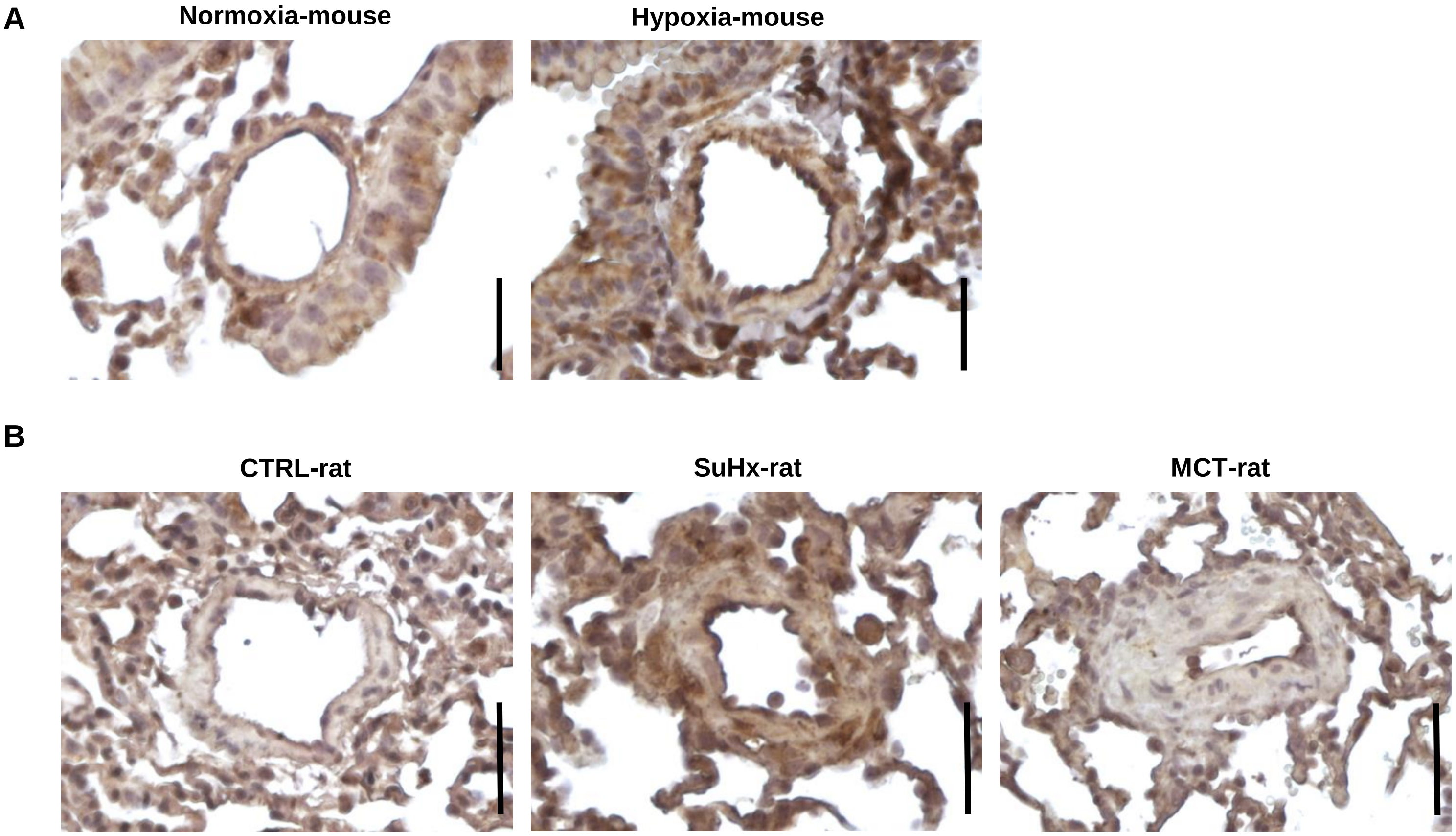

Supplementary Figure 2. ENO1 was elevated in the lungs of hypoxia-induced PH mice and SuHx-induced PH rats.

(A) Immunohistochemistry of ENO1 in the lung sections of control and hypoxia-induced PH mice (Scale bars, 50 μm). (B) Immunohistochemistry of ENO1 in the lung sections of control rats, SuHx-induced PH rats, and MCT-induced PH rats (Scale bars, 100 μm).

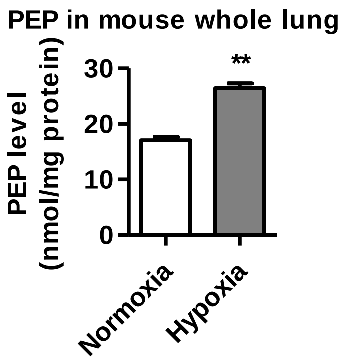

Supplementary Figure 3. PEP level is elevated in the whole lung tissue of hypoxia-induced PH mice.

PEP levels were measured in the whole lung tissue of hypoxia-induced PH mice and the control mice (n=4, ***P*<0.01). Data represent the mean ± SEM. Student t test were used to compare two groups.

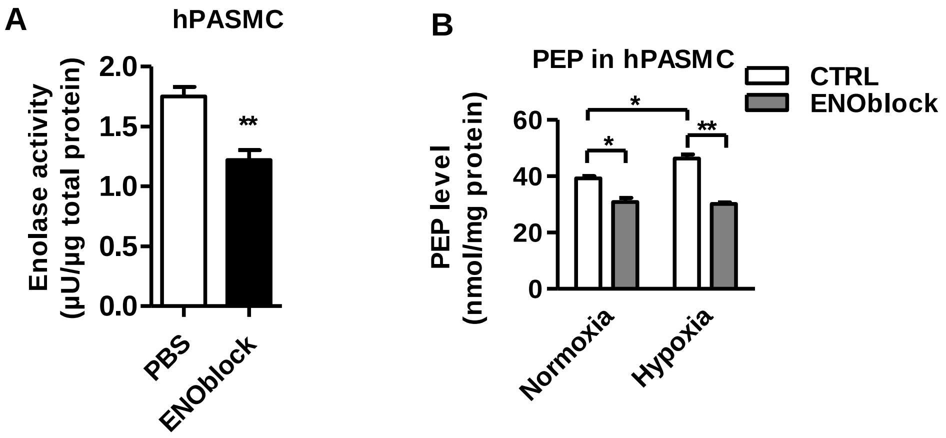

Supplementary Figure 4. ENOblock inhibits the enolase activity in PASMCs.

(A)We treated PASMC with 10 μM ENOblock for 8 h and measured the enolase activity (n=5). (B) We treated PASMC with 10 μM ENOblock, exposed them to normoxia or hypoxia for 8 h, and measured PEP levels in cell lysates (n=3-4). **P*<0.05, ***P*<0.01). Data represent the mean ± SEM. Student t test and one-way ANOVA were used to compare two and multiple groups. Bonferroni posttests were carried out after ANOVA.

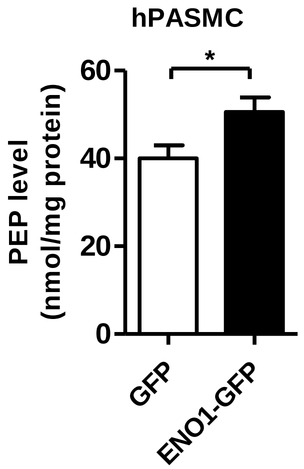

Supplementary Figure 5. ENO1 overexpression induces PEP levels in PASMC.

PASMCs were transfected with pCMV3-ENO1-GFP or control plasmid (GFP). 48 h after transfection, cells were collected and the intracellular PEP levels were measured (n=4-5, **P*<0.05). Data represent the mean ± SEM. Student t test were used to compare two groups.

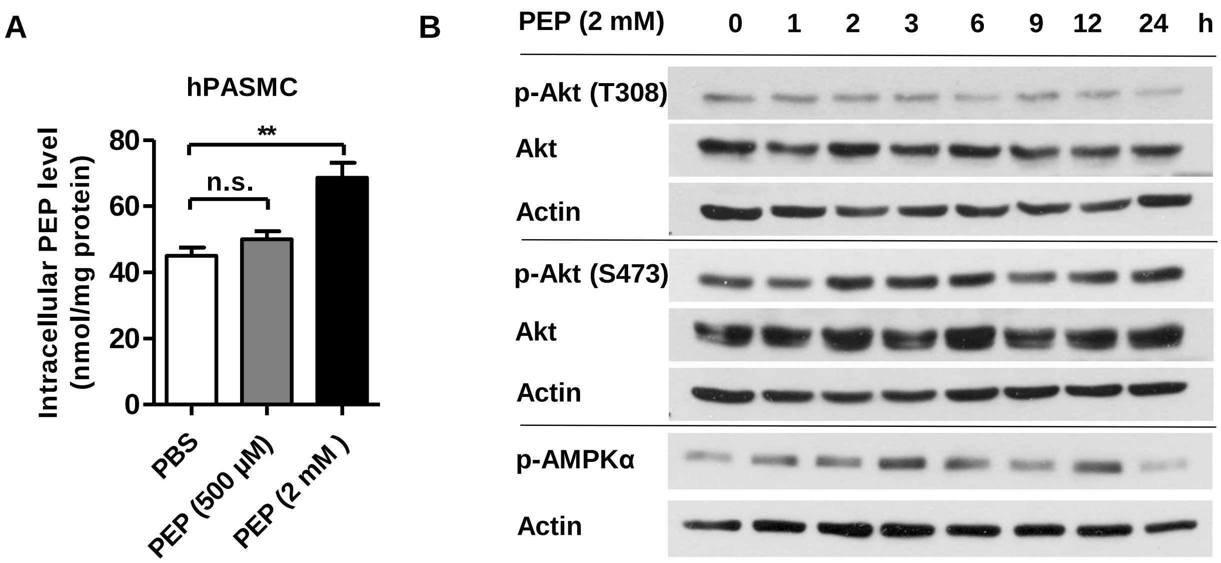

Supplementary Figure 6. The activation of the AMPK-Akt pathway is independent of PEP in PASMC.

(A) PASMCs were treated with 500 µM or 2 mM PEP for 24h, and the intracellular PEP level were measured afterwards (n=5, per group), **P<0.01, n.s. = non-significance. (B) PASMCs were treated with 2mM PEP for different periods of time and the levels of p-AMPKα and p-Akt were measured by Western blotting in the cell lysate. Data represent the mean ± SEM. Student t test and one-way ANOVA were used to compare two and multiple groups. Bonferroni posttests were carried out after ANOVA.

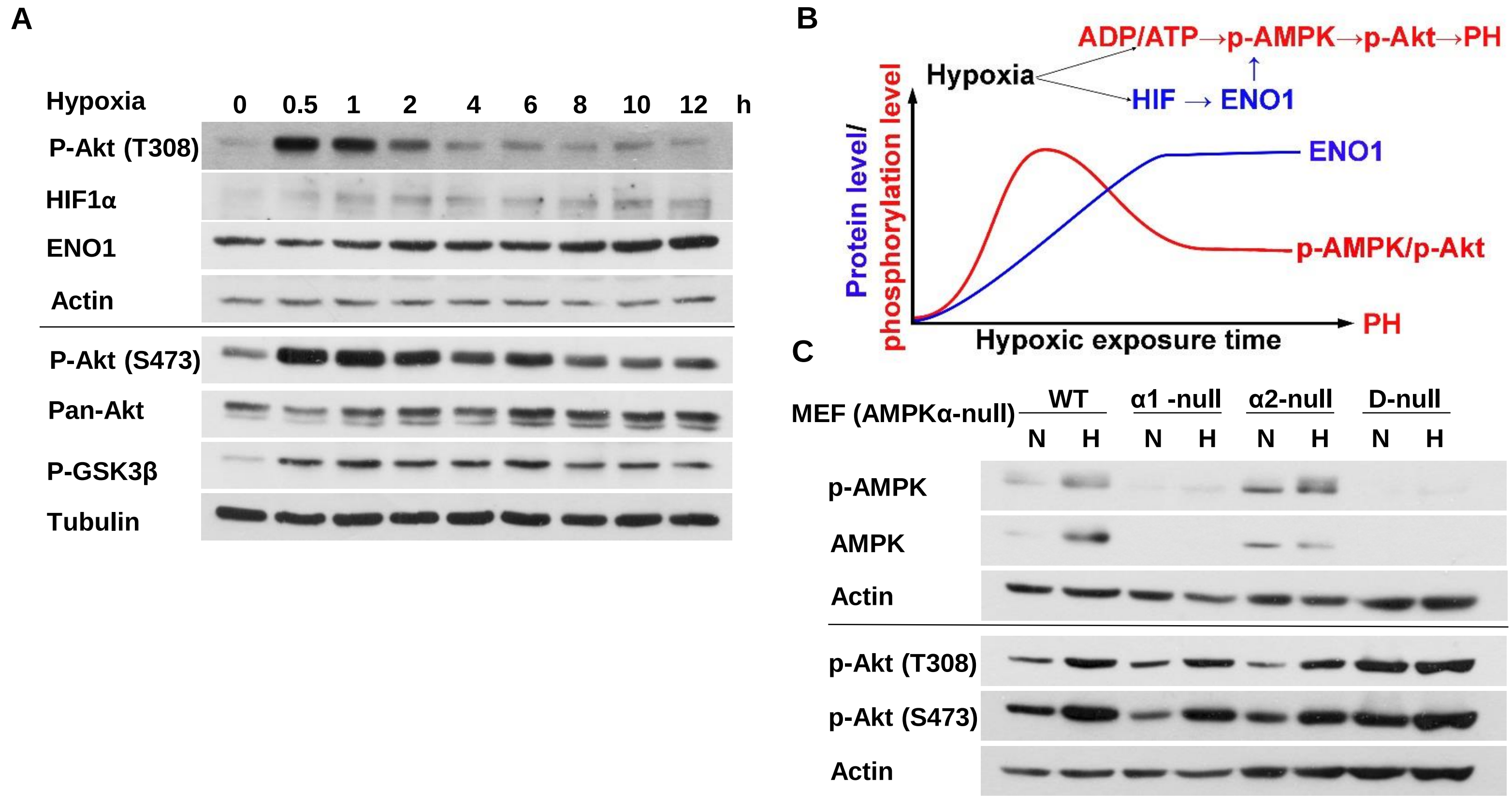

Supplementary Figure 7. ENO1 is responsible for the sustained activation of AMPK-Akt-GSK3β axis during hypoxia.

(A) PASMCs were treated with hypoxia for different periods of time, and the key proteins in the ENO1-AMPK-Akt-GSK3β cascade were measured by Western blotting in the cell lysate. (B) A diagrammatic sketch indicating the mechanism of transient and continuous activation of ENO1-AMPK-Akt-GSK3β cascade during hypoxia. (C) Wild-type (WT), AMPKα1–null, and AMPKα2–null MEFs were treated with hypoxia for 30 min, and the key proteins in the AMPK-Akt cascade were measured by Western blotting.

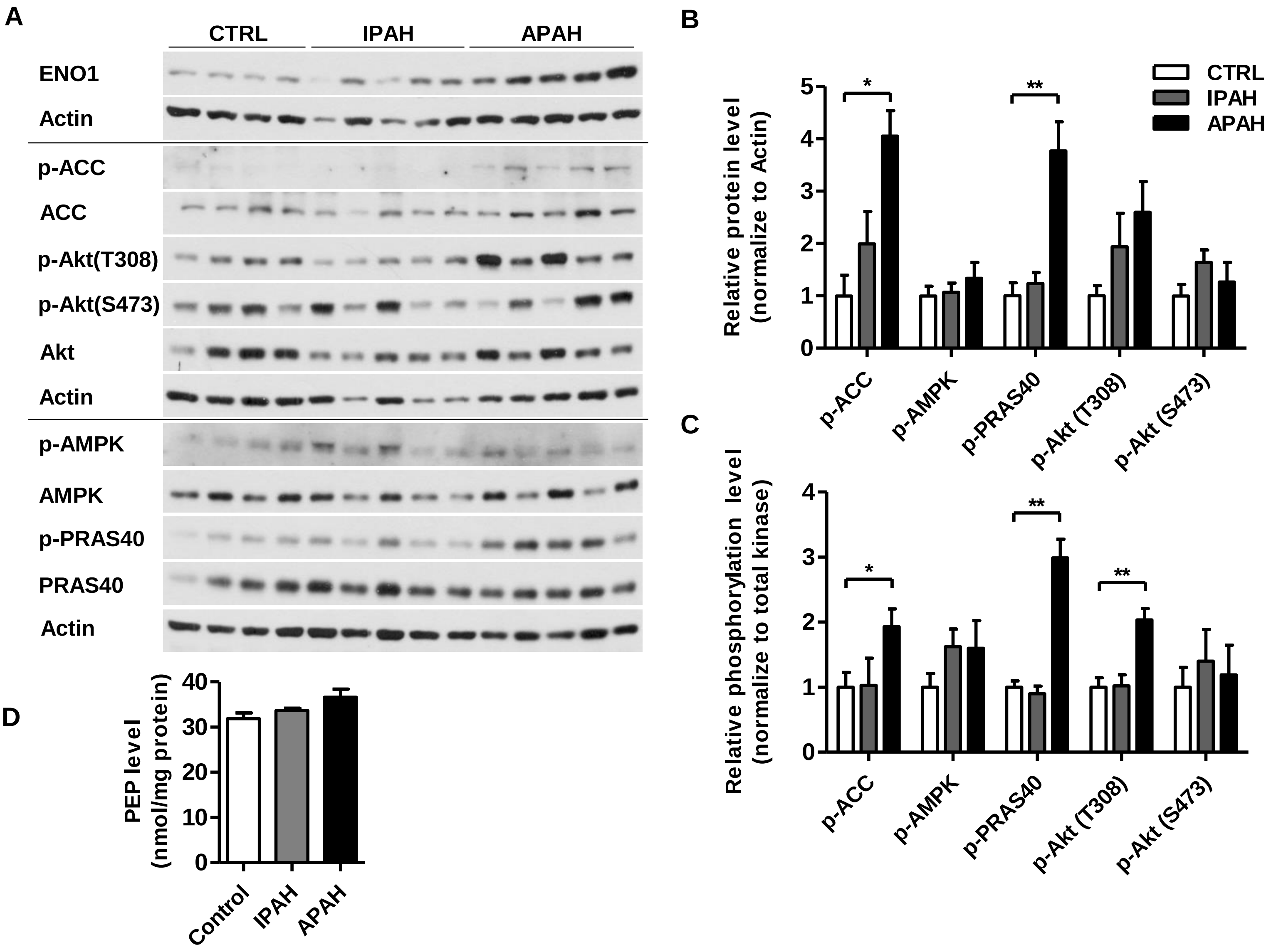

Supplementary Figure 8. The AMPK-Akt pathway is activated in APAH-PASMC independent of PEP.

(A) Western blotting images and (B,C) normalized quantification (to Actin or its total corresponding kinase, respectively) of AMPK/Akt pathway phosphorylated proteins in PASMC isolated from control donors and PAH patients (n=4-5 per group, *P<0.05, **P<0.01). (D) The intracellular PEP levels were measured in these PASMC samples. Data represent the mean ± SEM. Student t test and one-way ANOVA were used to compare two and multiple groups. Bonferroni posttests were carried out after ANOVA.

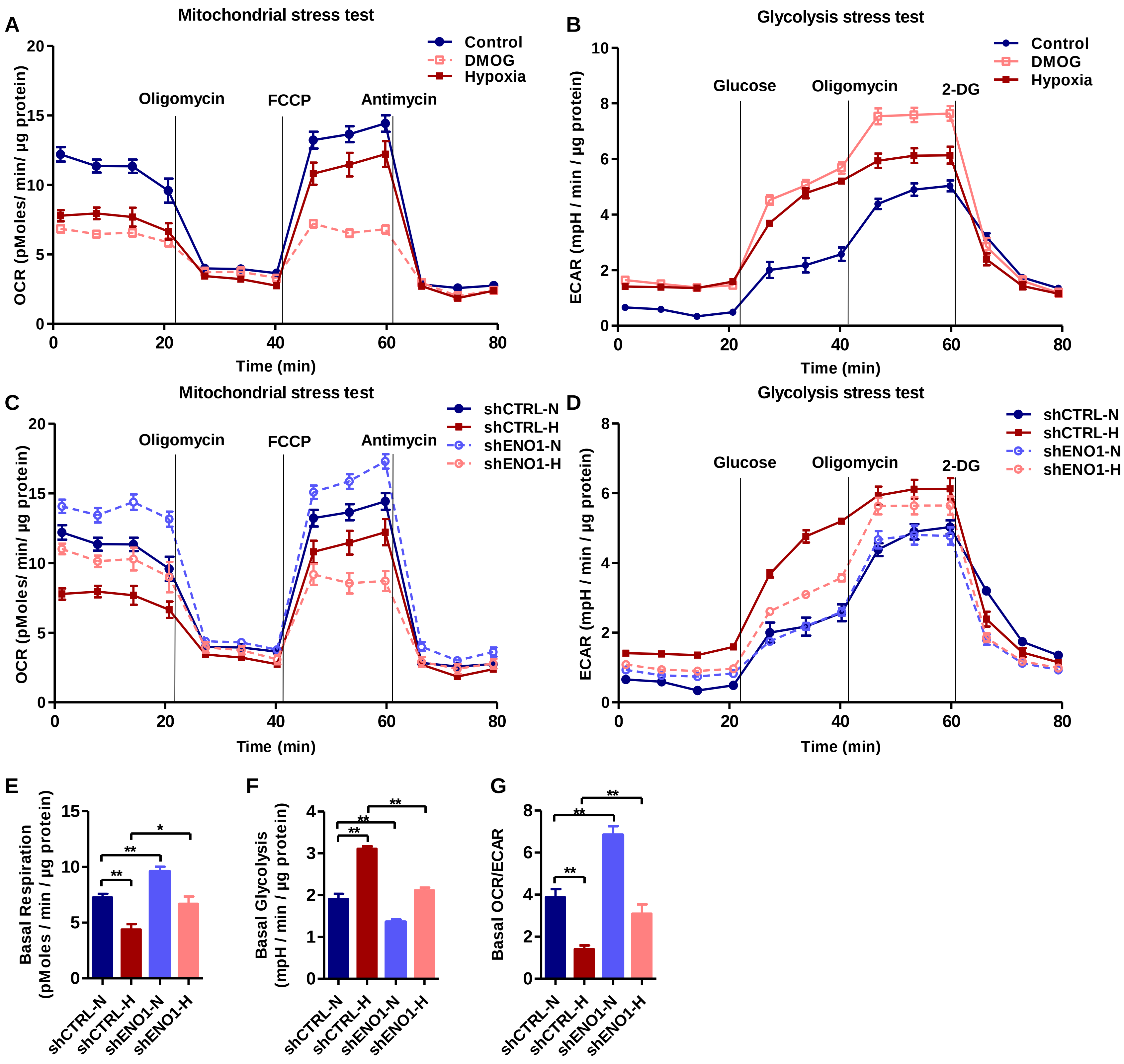

Supplementary Figure 9. Silencing of ENO1 reverses hypoxia-induced metabolic shift in PASMC.

PASMC (shCTRL and shENO1) with a relatively long-term of hypoxia (2% O2) for 48 h. After the treatment, we seeded the cells in the plates for 4 hours to allow the cells attached to the plate and formed a monolayer. DMOG treatment group was also included as a positive control, and the DMOG was kept in the cultural media during the tests. (A) The OCR levels were measured using the mitochondrial stress test (n=4-5 per group), and (B) the ECAR levels were measured using the glycolysis stress test (n=4-5 per group), showing the effects of hypoxia and DMOG. (C) The OCR levels measured by the mitochondrial stress test and (D) the ECAR levels were measured using the glycolysis stress test, showing the effects of ENO1 silencing on hypoxia-mediated metabolic shift. The (E) basal respiration level, (F) basal glycolytic level, and (G) basal OCR/ECAR were calculated accordingly. *P<0.05, **P<0.01, n.s. = non-significance. Data represent the mean ± SEM. Student t test and one-way ANOVA were used to compare two and multiple groups. Bonferroni posttests were carried out after ANOVA.

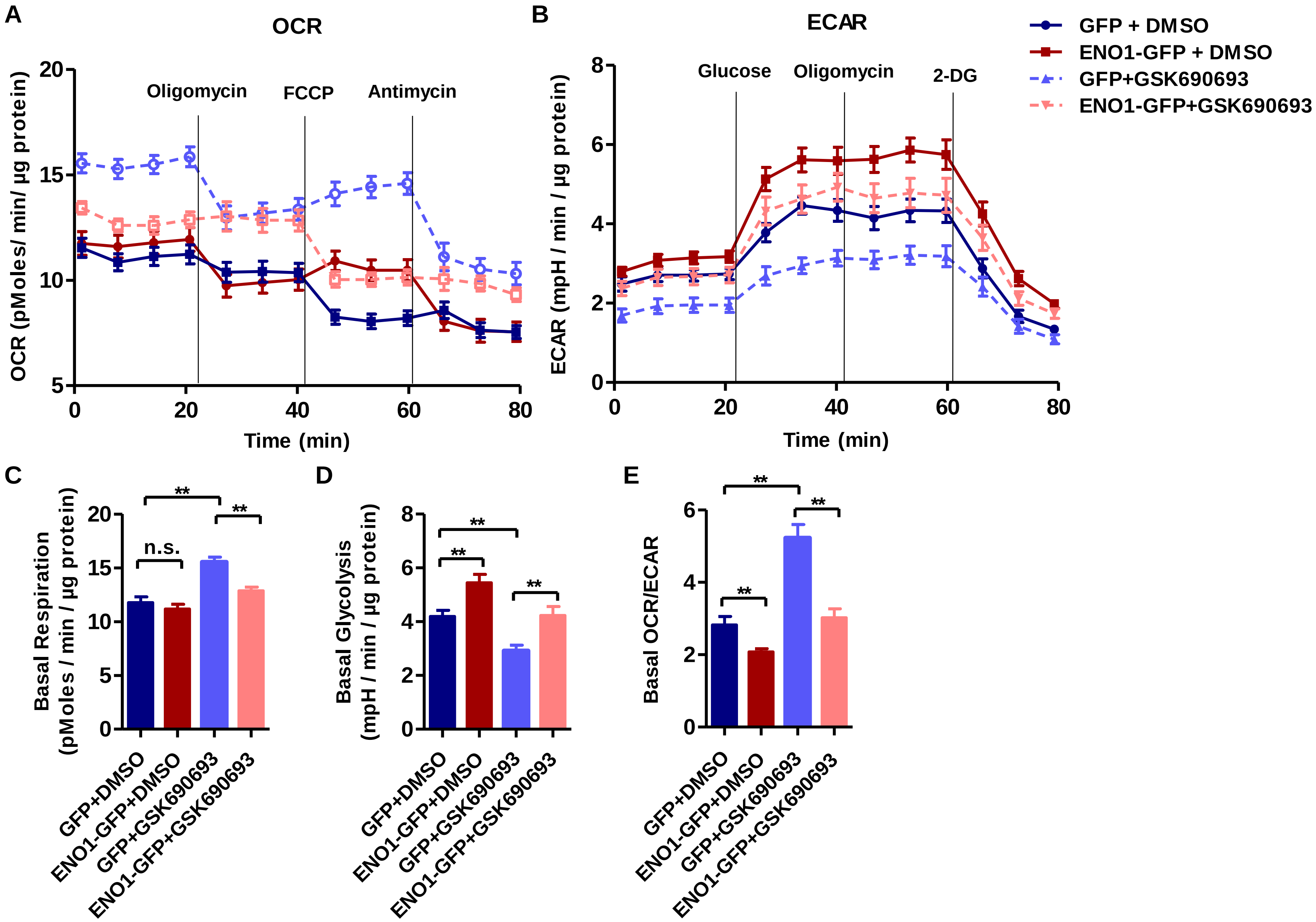

Supplementary Figure 10. ENO1 mediates the metabolic shift to glycolysis partly via Akt activation in PASMC.

PASMC was transfected with pCMV3-ENO1-GFP. After 48 h, we seed the cells in the plates and treated with 1 μM GSK690693 for 12 h, followed by the Seahorse tests. (A) The OCR levels were measured using the mitochondrial stress test (n=5-8 per group), and (B) the ECAR levels were measured using the glycolysis stress test (n=5-8 per group) showing the effects of ENO1-overexpression and Akt inhibition on cell metabolism. The (C) basal respiration level, (D) basal glycolytic level, and (E) basal OCR/ECAR were calculated accordingly. **P<0.01, n.s. = non-significance. Data represent the mean ± SEM. Student t test and one-way ANOVA were used to compare two and multiple groups. Bonferroni posttests were carried out after ANOVA.

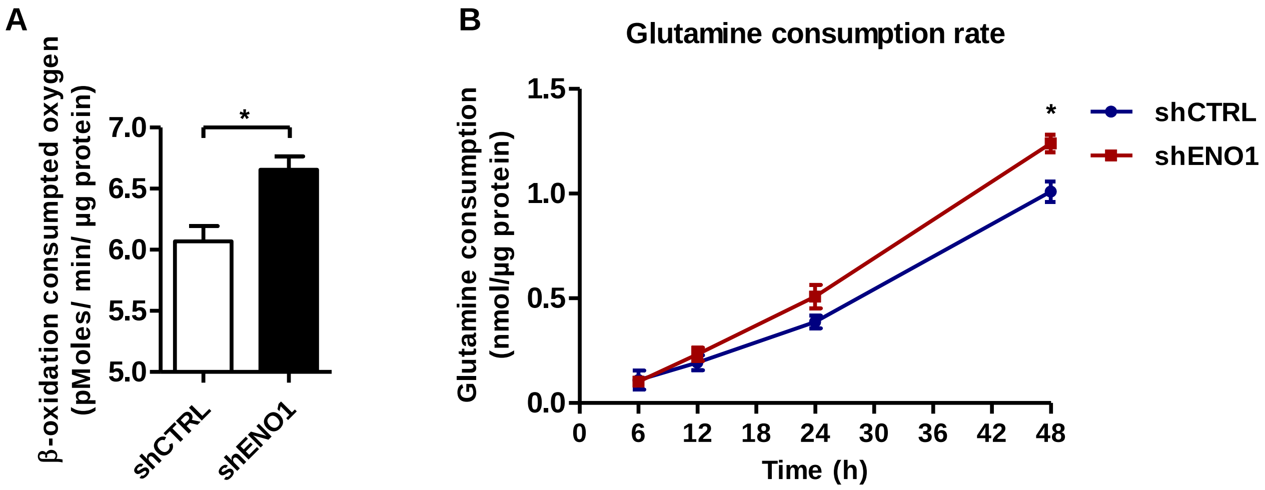

Supplementary Figure 11. Silencing of ENO1 promotes β-oxidation and glutamine consumption level in PASMC.

(A) PASMC were kept in glucose-depleted medium contains Oleate as energy source for 1h before the basal O_2 consumption levels were measured using the Seahorse system. (B) PASMC were cultured in starved medium and the glutamine concentration in cell culture media after different incubation time were measured thus the glutamine consumption levels can be calculated. *P<0.05. Data represent the mean ± SEM. Student t test and one-way ANOVA were used to compare two and multiple groups. Bonferroni posttests were carried out after ANOVA.

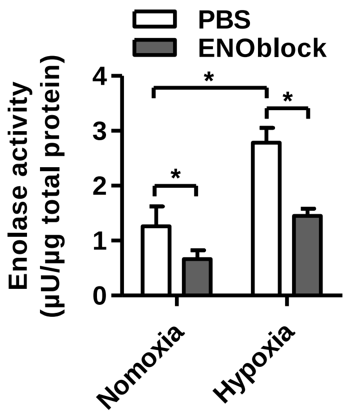

Supplementary Figure 12. ENOblock treatment inhibits enolase activity in the mouse whole lung tissue.

The enolase activities were measured in the lysate of whole lung tissues isolated from the ENOblock treated mice in both normoxic and hypoxic conditions (n=3, *P<0.05). Data represent the mean ± SEM. Student t test and one-way ANOVA were used to compare two and multiple groups. Bonferroni posttests were carried out after ANOVA.

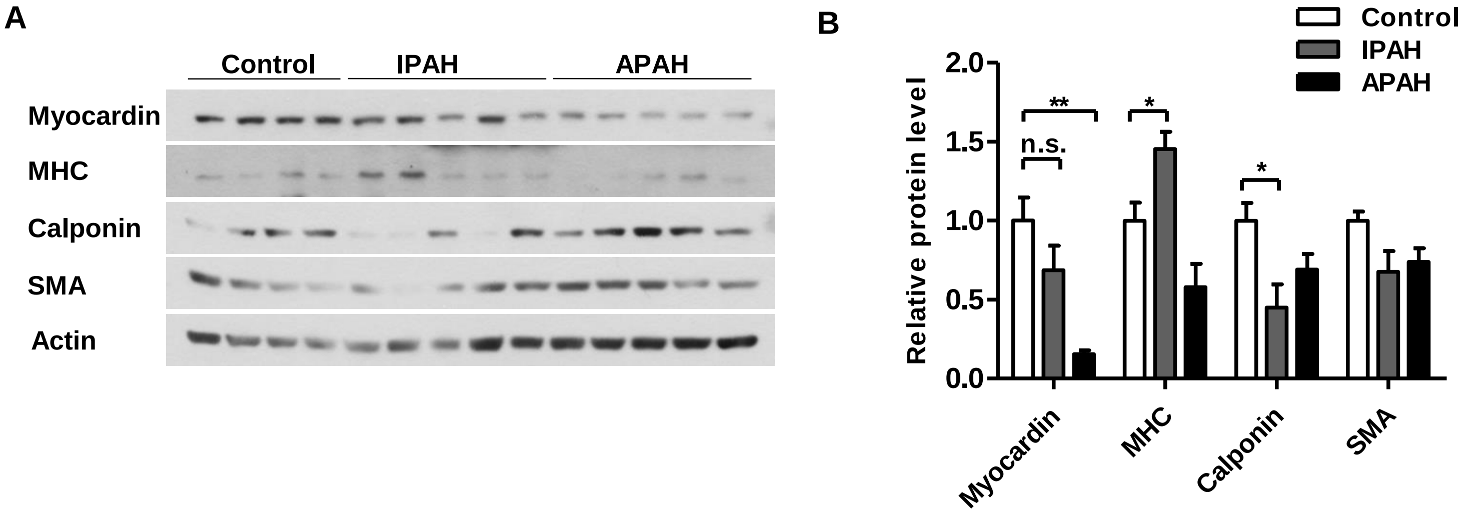

Supplementary Figure 13. Myocardin is down-regulated in APAH-PASMC*.*

(A) Western blotting images and (B) normalized quantification of SMC contractile proteins (Myocardin, MHC, Calponin, and SMA,) in PASMCs isolated from control donors and PAH patients (n=4-5 per group, *P<0.05, **P<0.01). Data represent the mean ± SEM. Student t test and one-way ANOVA were used to compare two and multiple groups. Bonferroni posttests were carried out after ANOVA.

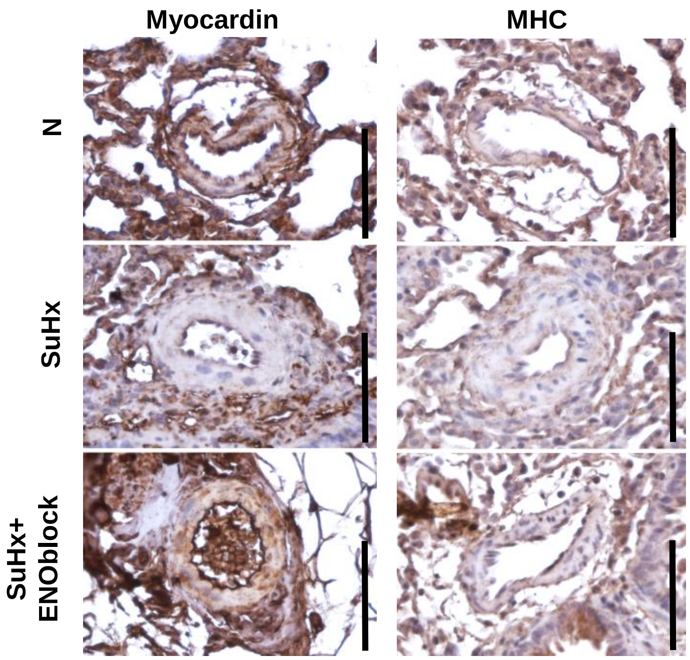

Supplementary Figure 14. Inhibition of ENO1 reversed Sugen/hypoxia-induced PASMC de-differentiation in rats. Immunohistochemistry staining of Myocardin and MHC in a pulmonary artery of lung sections of Sugen/Hypoxia-induced PH rats and treated with ENOblock (Scale bars, 100 μm).

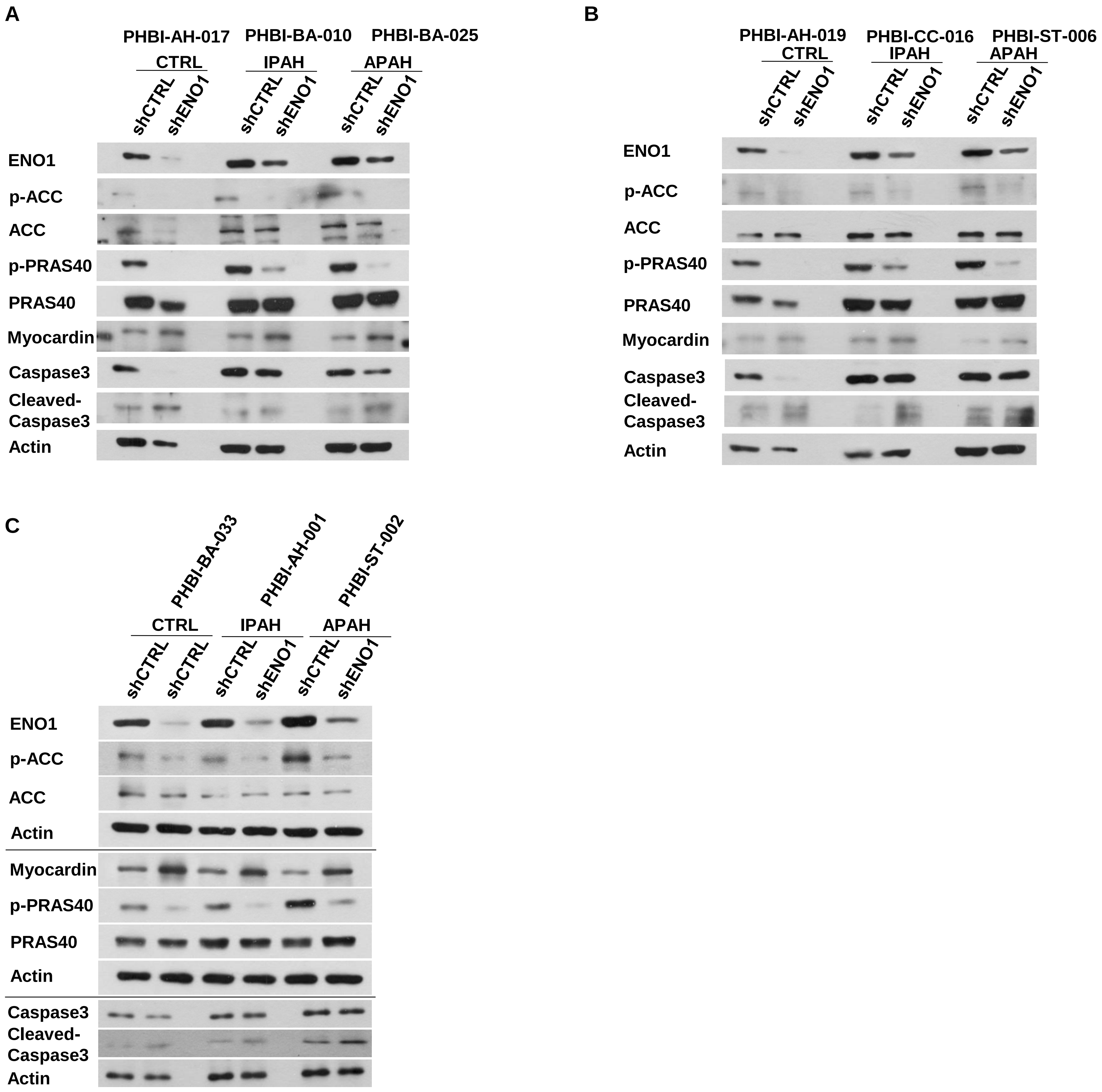
A
PHBI-AH-017
PHBI-BA-010
PHBI-BA-025
CTRL
IPAH
APAH
shCTRL
shENO1
ENO1
p-ACC
ACC
p-PRAS40
PRAS40
Myocardin
Caspase3
Cleaved-Caspase3
Actin
B
PHBI-AH-019
PHBI-CC-016
PHBI-ST-006
CTRL
IPAH
APAH
shCTRL
shENO1
ENO1
p-ACC
ACC
p-PRAS40
PRAS40
Myocardin
Caspase3
Cleaved-Caspase3
Actin
C
PHBI-BA-033
PHBI-AH-001
PHBI-ST-002
CTRL
IPAH
APAH
shCTRL
shCTRL
shCTRL
shENO1
shCTRL
shENO1
ENO1
p-ACC
ACC
Actin
Myocardin
p-PRAS40
PRAS40
Actin
Caspase3
Cleaved-Caspase3
Actin

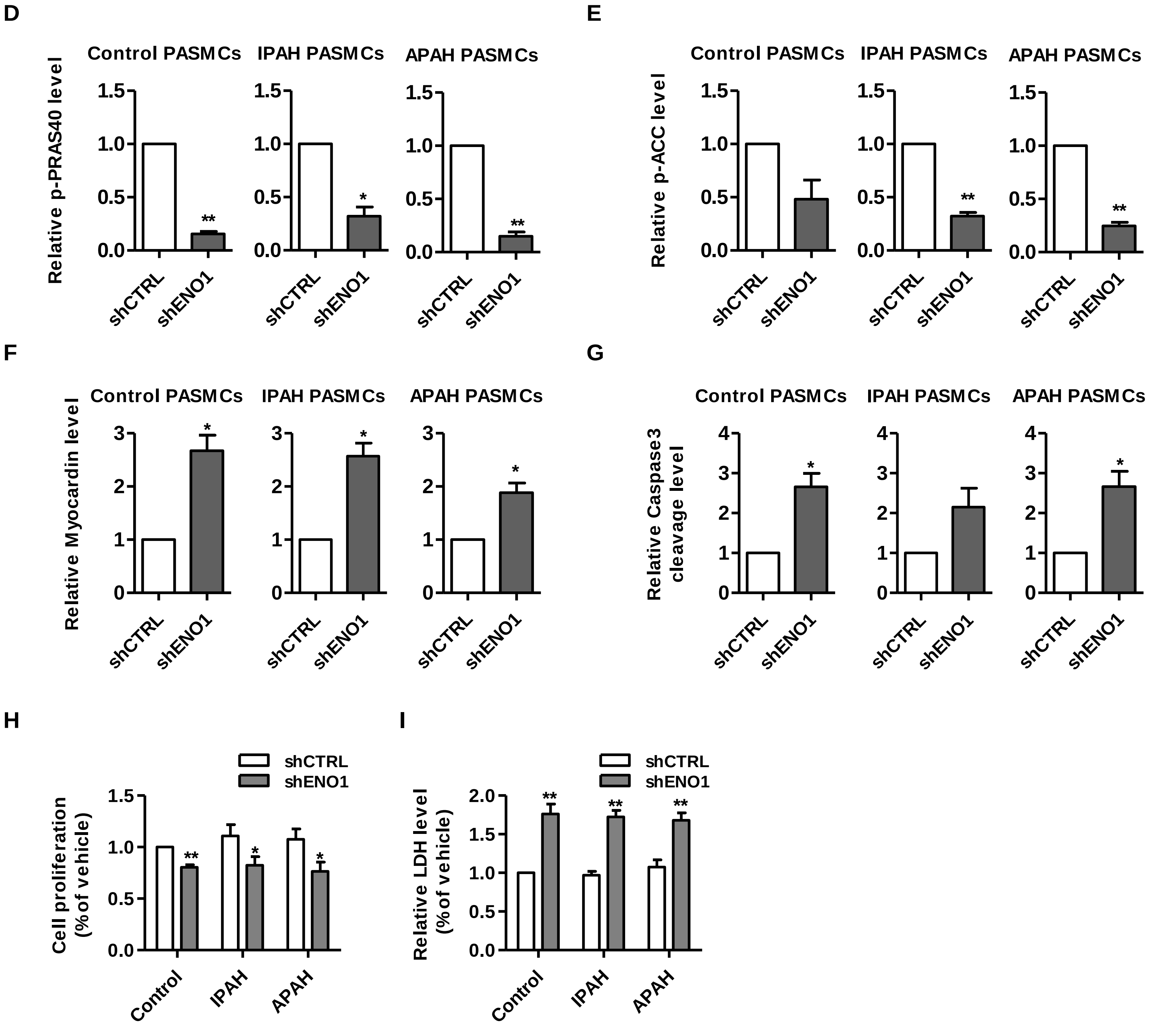

Supplementary Figure 15. Silencing of ENO1 simultaneously inhibits the AMPK/Akt pathway and elevates Myocardin levels and Caspase 3 cleavage in PASMC.

(A-C)Three lines of PASMC isolated from each group of control donors and PAH patients were transfected with lentivirus containing an shRNA against ENO1. The p-ACC, p-PRAS40, Myocardin levels and Caspase 3 cleavage were measured by Western-blotting and quantified in D-G, respectively. (H) The cell proliferation and (I) cell death level were measured by the BrdU assay and LDH assay. n=3 per group, *P<0.05, **P<0.01. Student t test and one-way ANOVA were used to compare two and multiple groups. Bonferroni posttests were carried out after ANOVA.

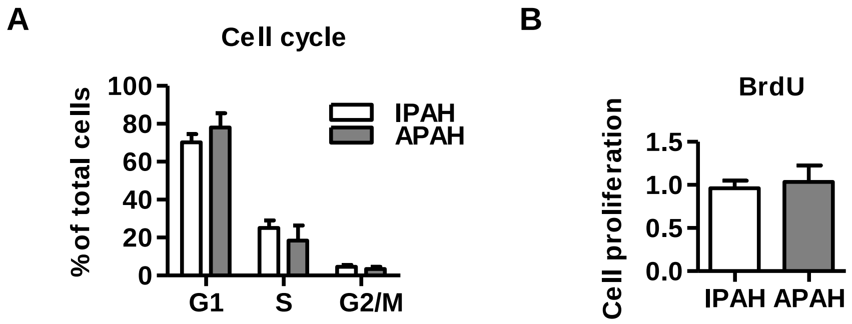

Supplementary Figure 16. Cell proliferation between IPAH-PASMC and APAH-PASMC. Human PASMC samples (IPAH PASMC, n=13, APAH PASMC, n=6) were subjected to (A) cell cycle analysis and the (B) BrdU incorporation assay.

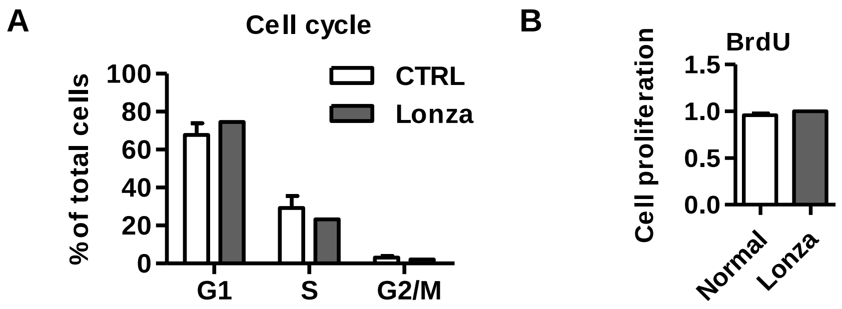

Supplementary Figure 17. Cell proliferation between human PASMC purchased from Lonza and normal PASMC from donors.

Human PASMC purchased from Lonza and samples from control donor (n=8) were subjected to (A) cell cycle analysis and (B)the BrdU incorporation assay.

Figure 1A

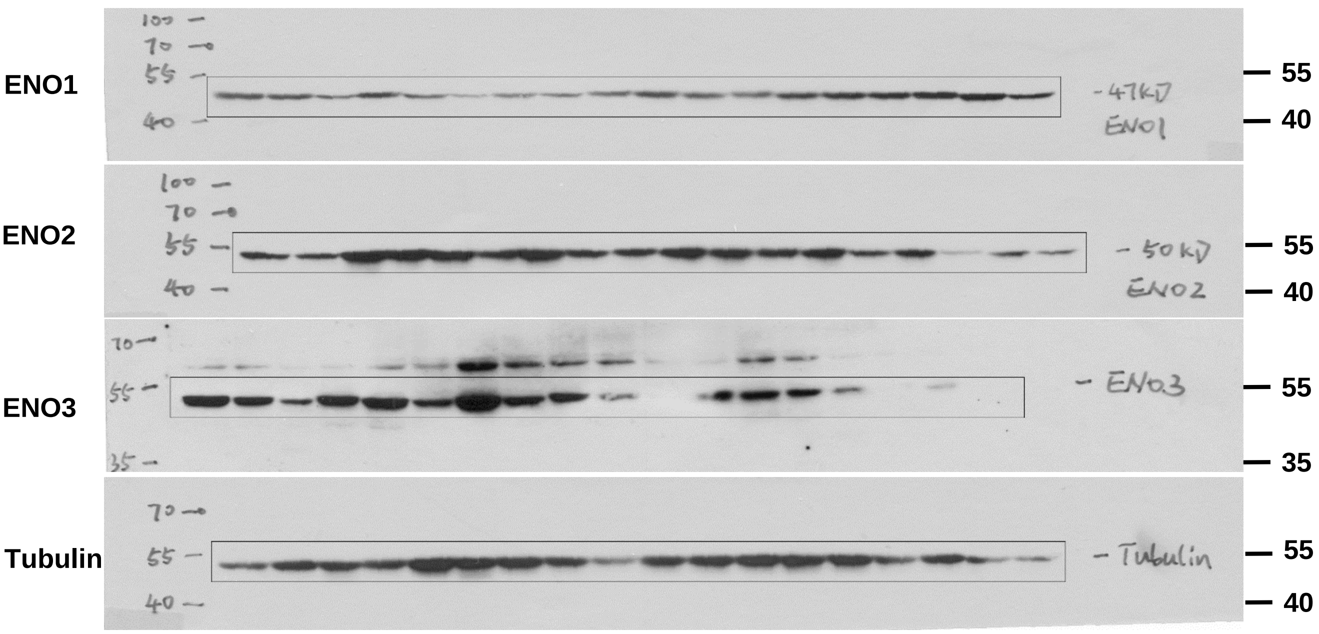

Figure 1E

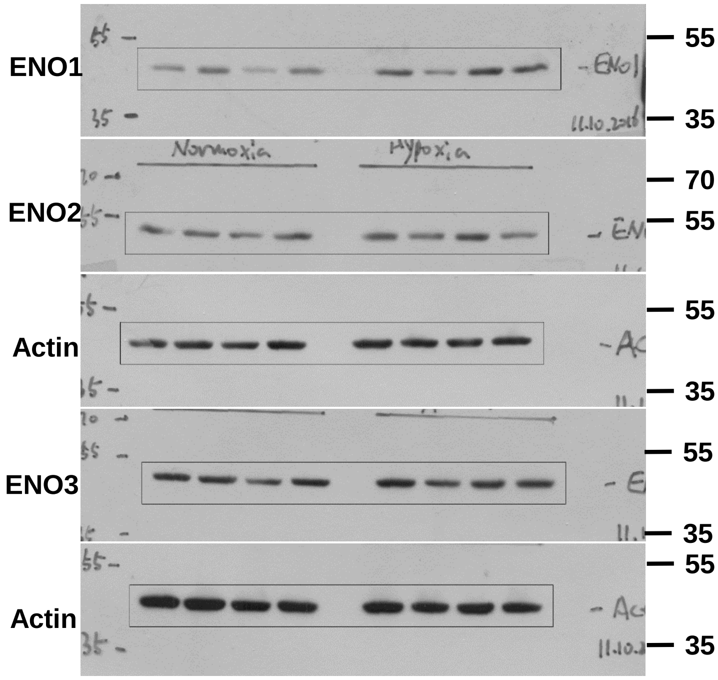

Figure 1H

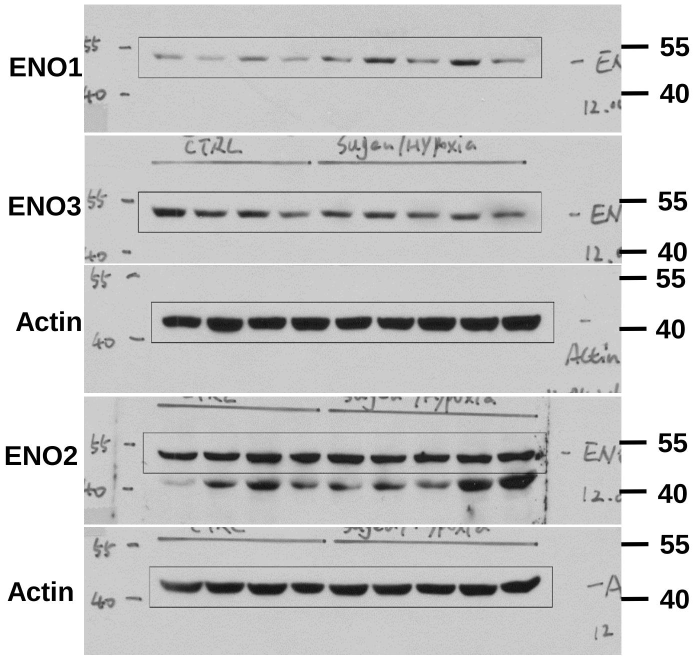

Supplementary Figure 18. Uncropped Western blots for Figure 1A, E, and H.

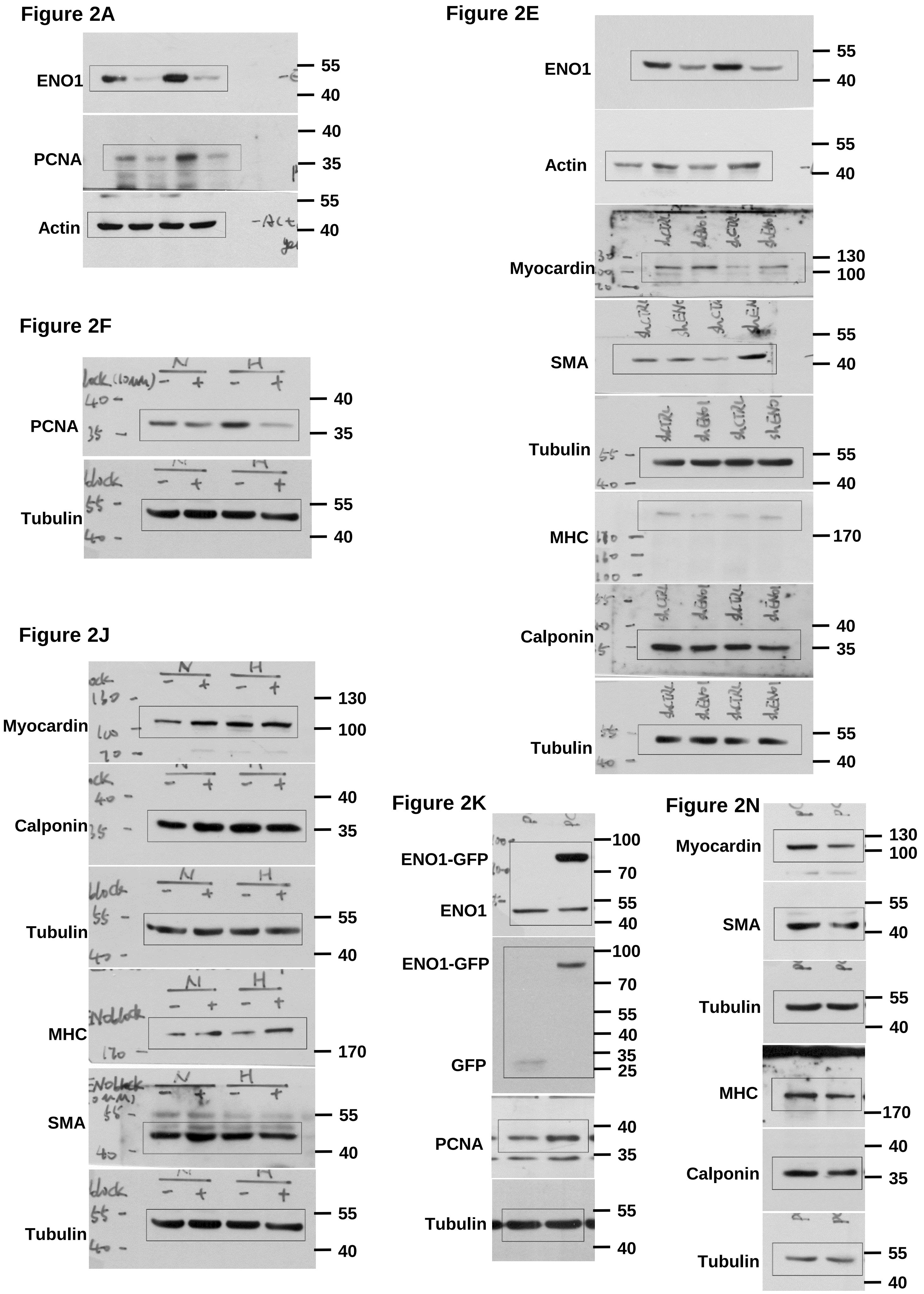

Supplementary Figure 19. Uncropped Western blots for Figure 2A, E, F, J, K, and N.

Figure 3A

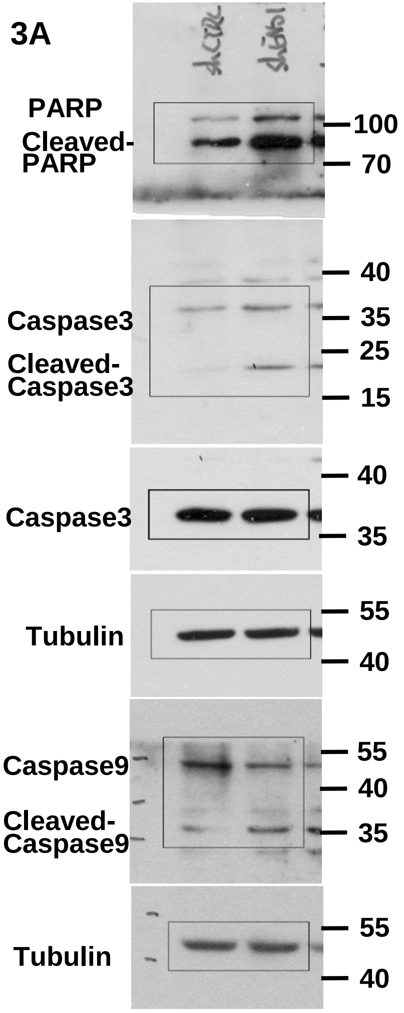

Figure 3E

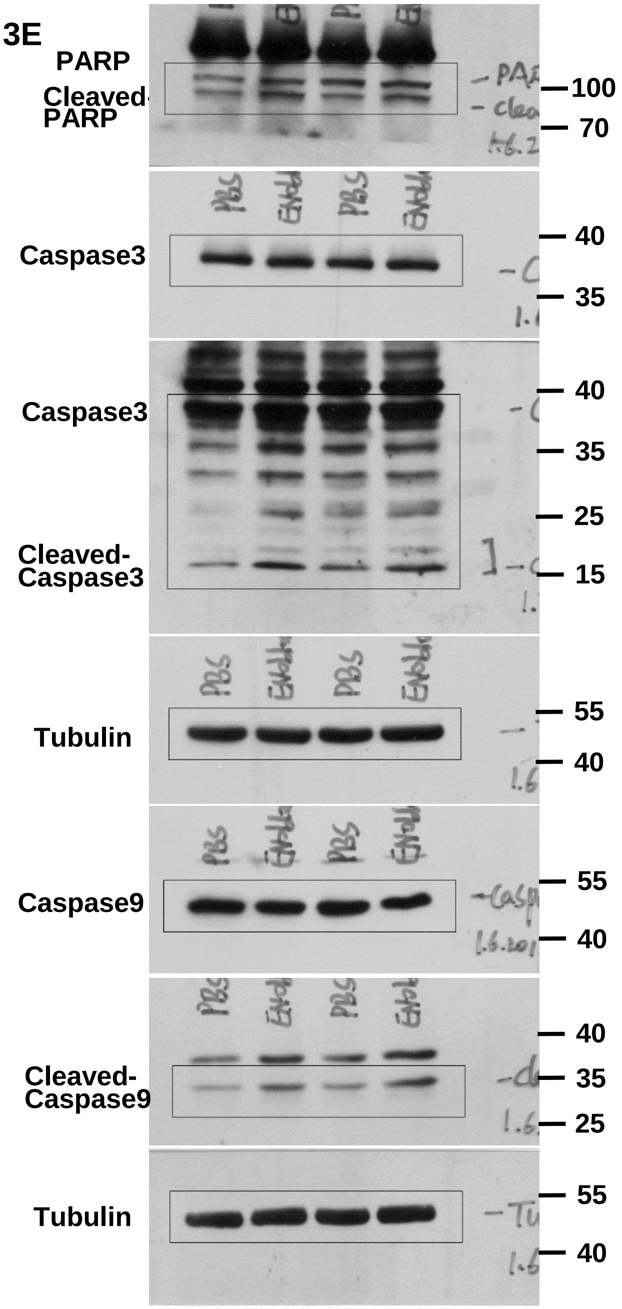

Figure 3I

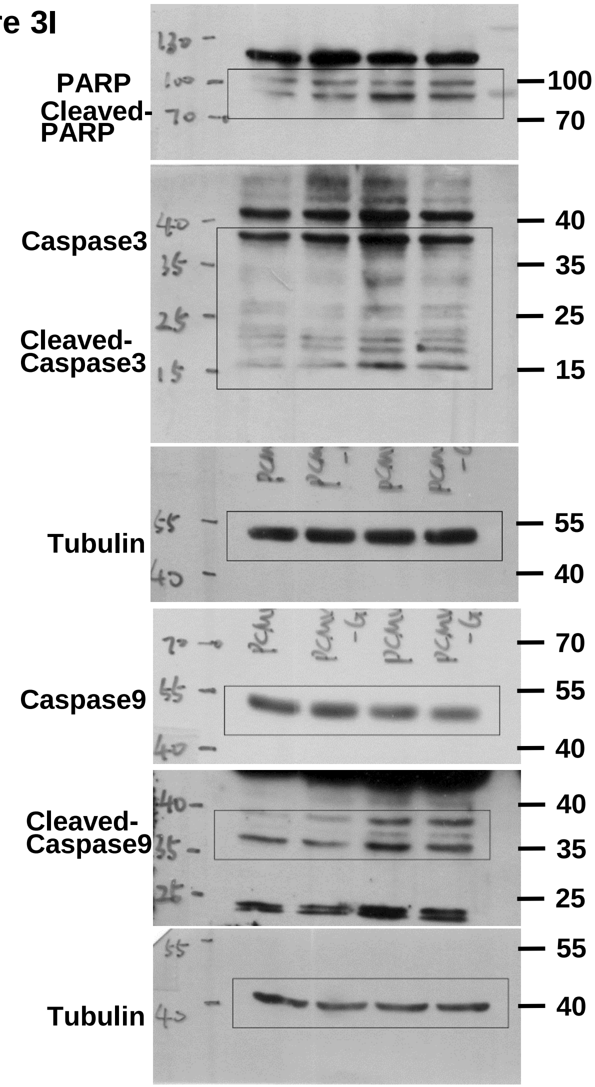

Supplementary Figure 20. Uncropped Western blots for Figure 3A, E, and i.

Figure 4A

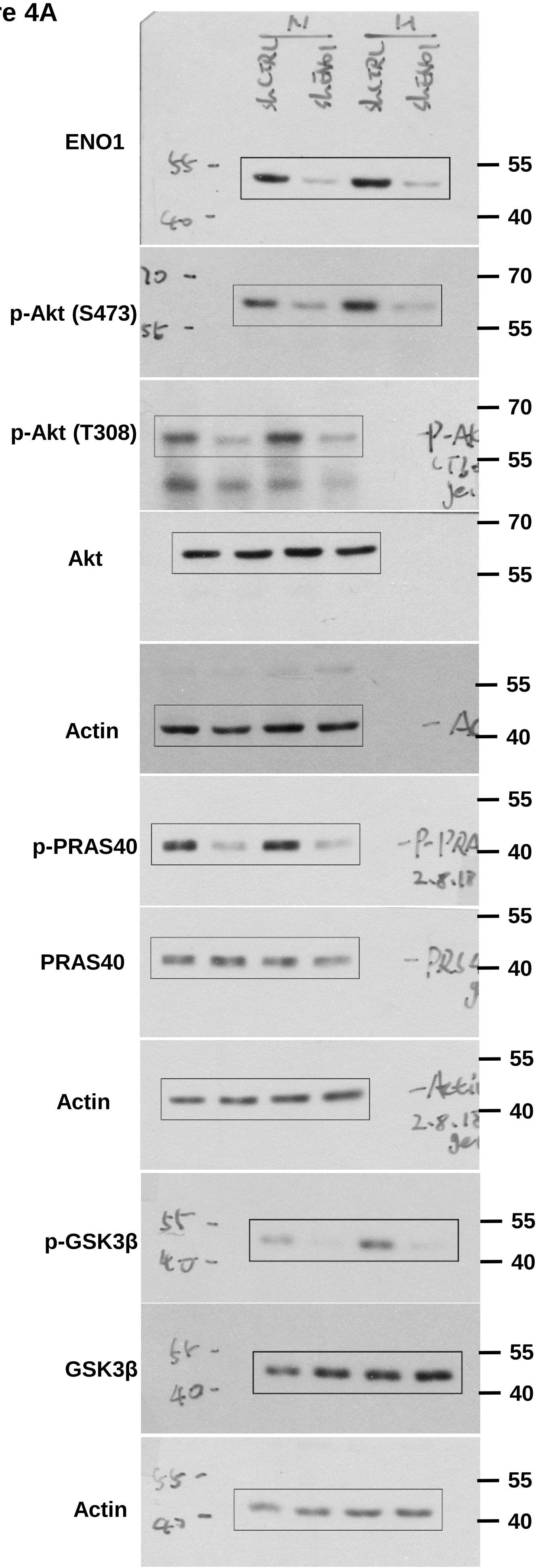

Figure 4B

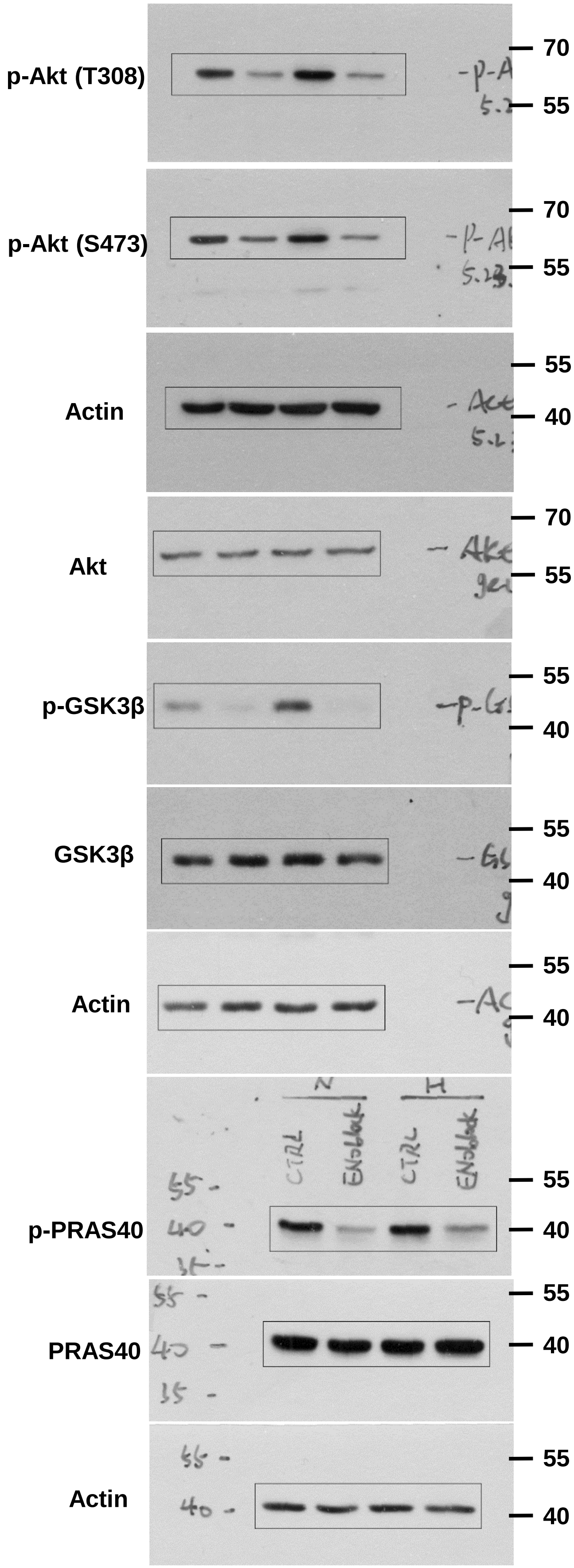

Supplementary Figure 21. Uncropped Western blots for Figure 4A, and B.

Figure 4C

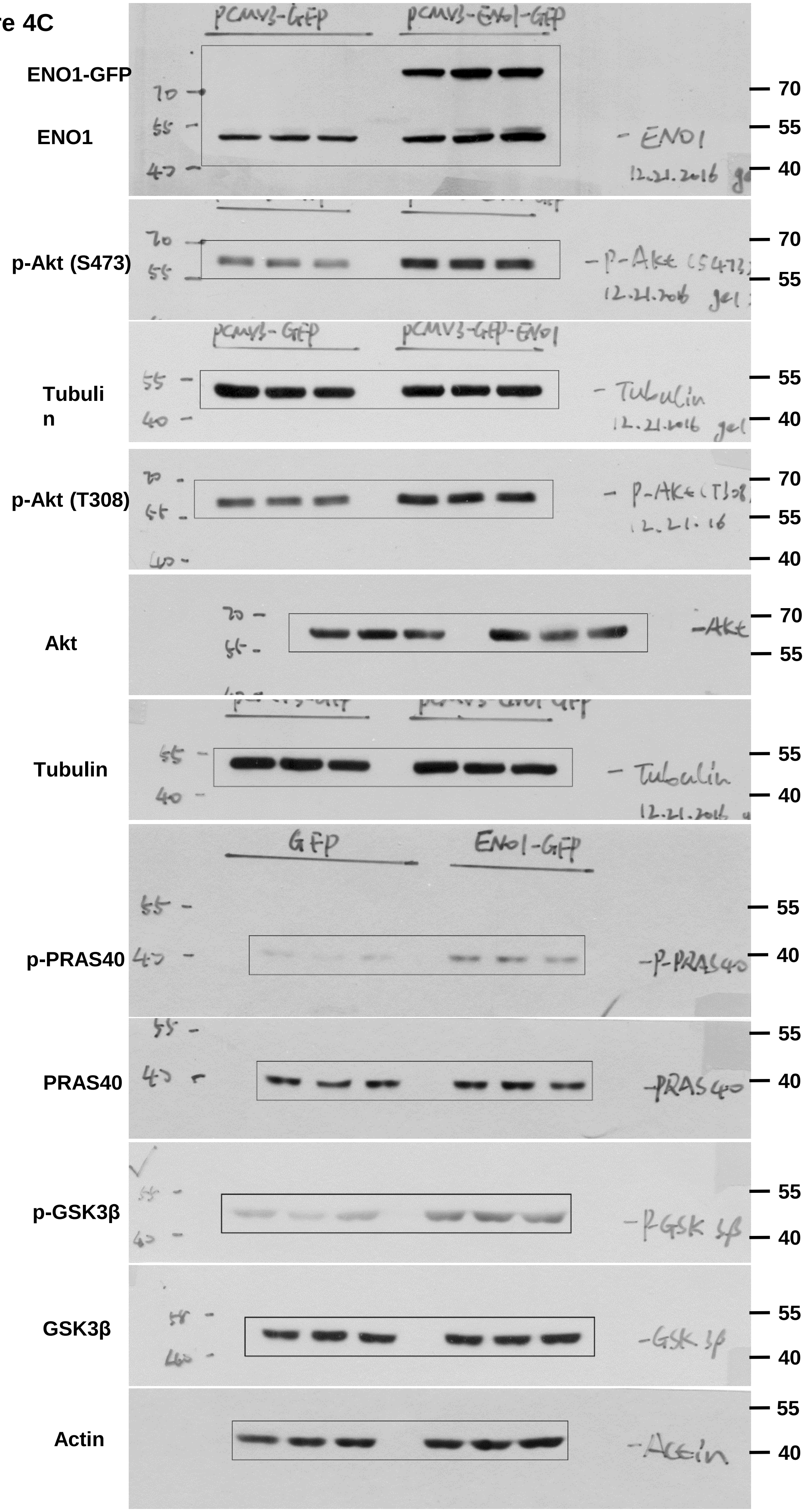

Supplementary Figure 22. Uncropped Western blots for Figure 4C.

Figure 4E

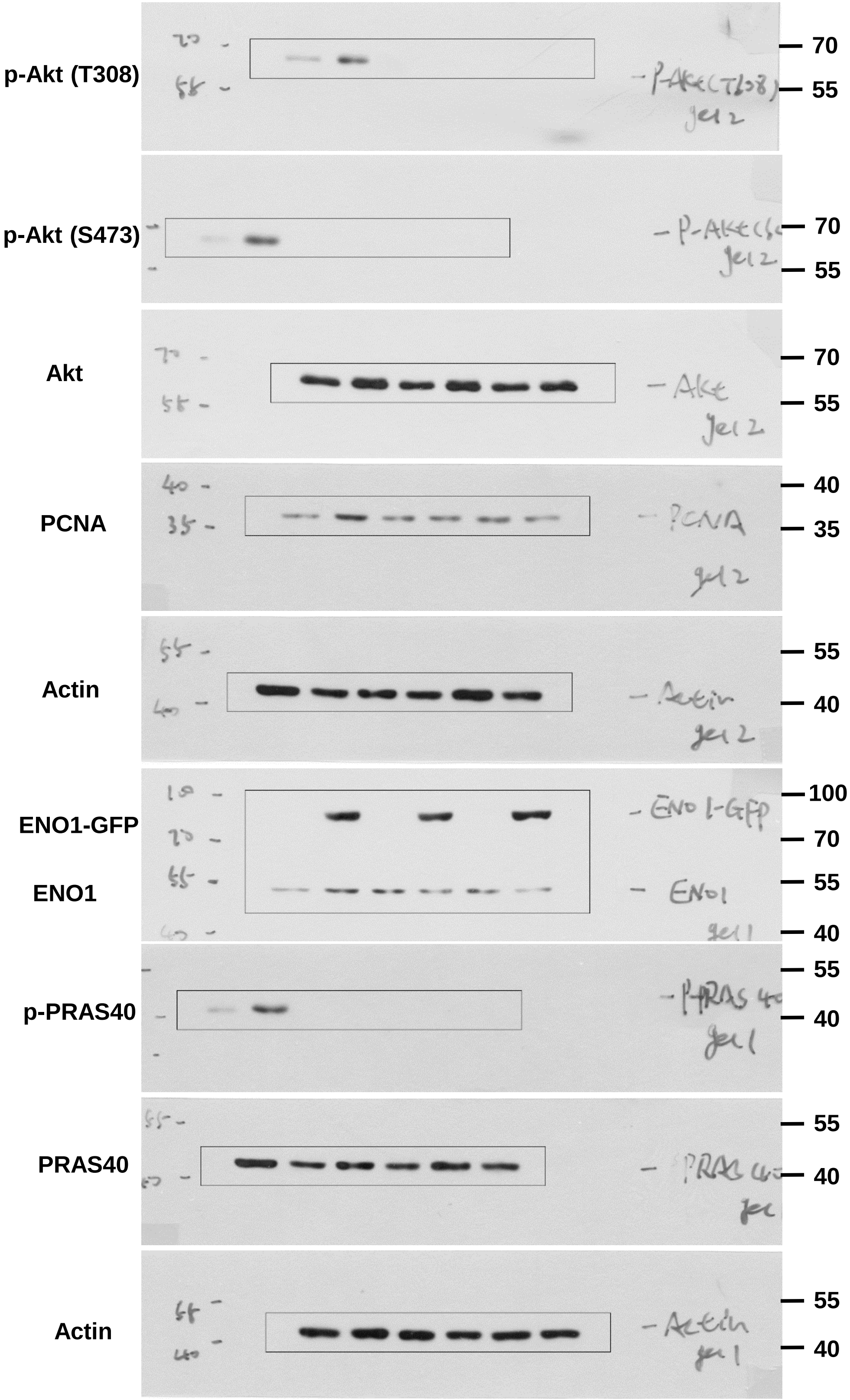

Supplementary Figure 23. Uncropped Western blots for Figure 4E.

Figure 5A

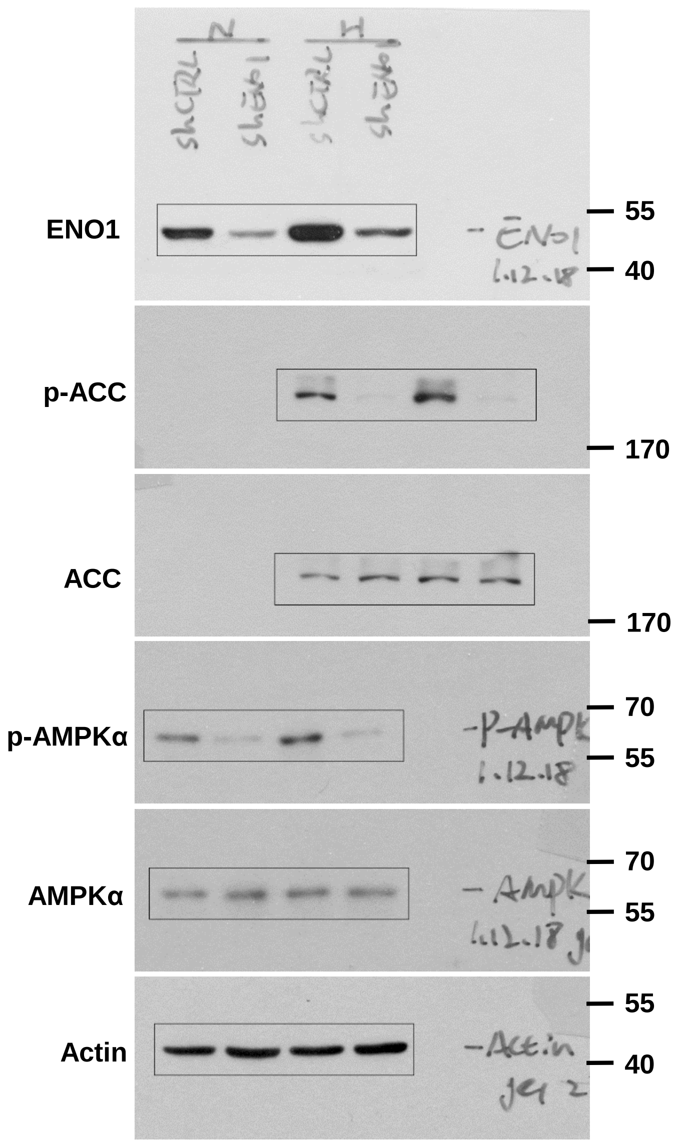

Figure 5C

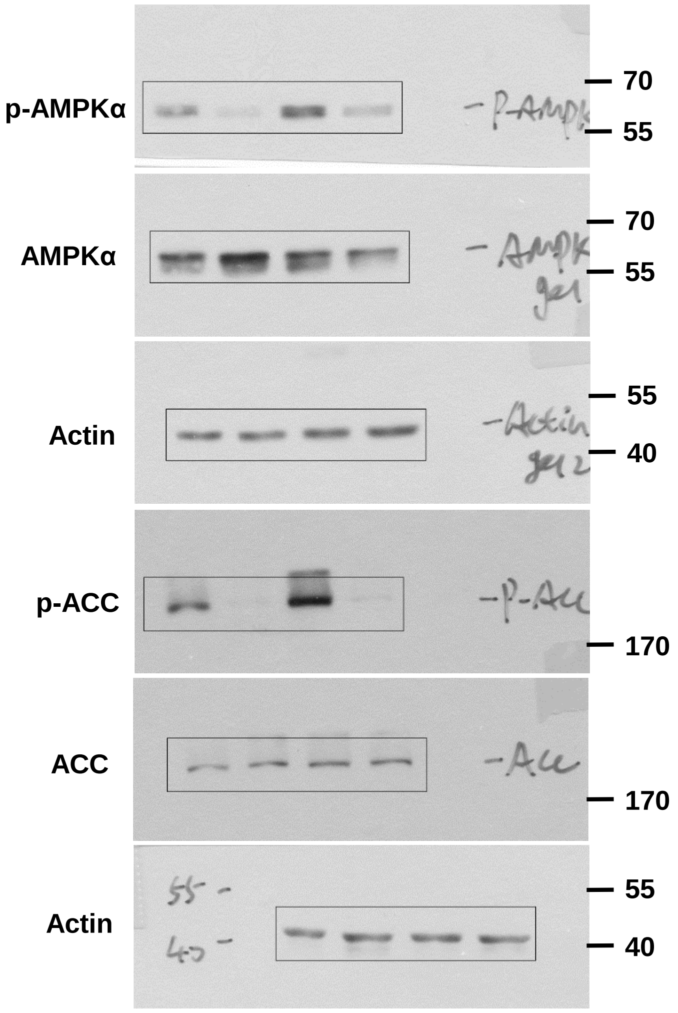

Supplementary Figure 24. Uncropped Western blots for Figure 5A, and C.

Figure 5D

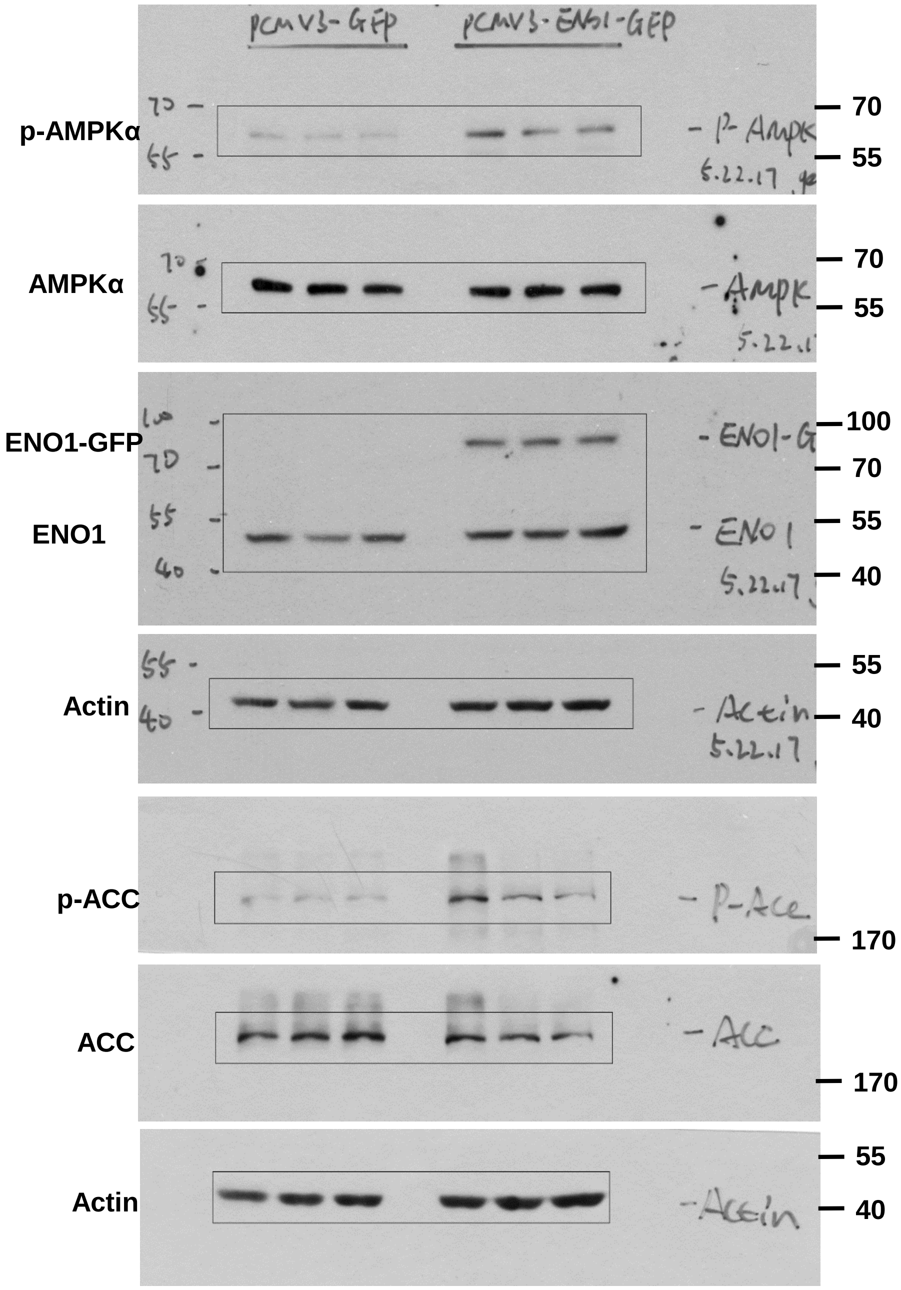

Figure 5F

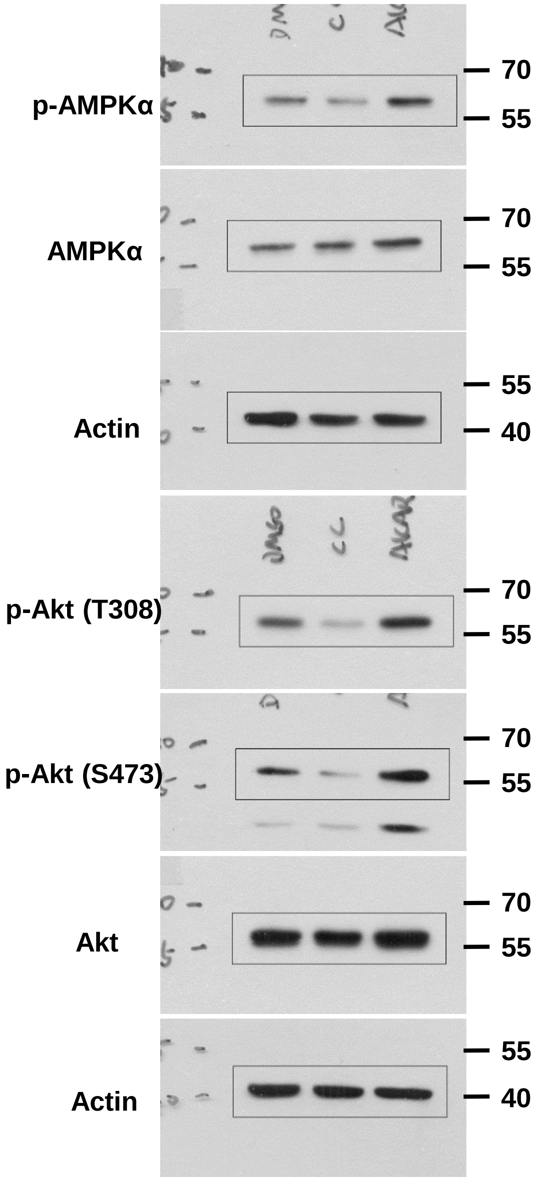

Supplementary Figure 25. Uncropped Western blots for Figure 5D, and F.

Figure 5G

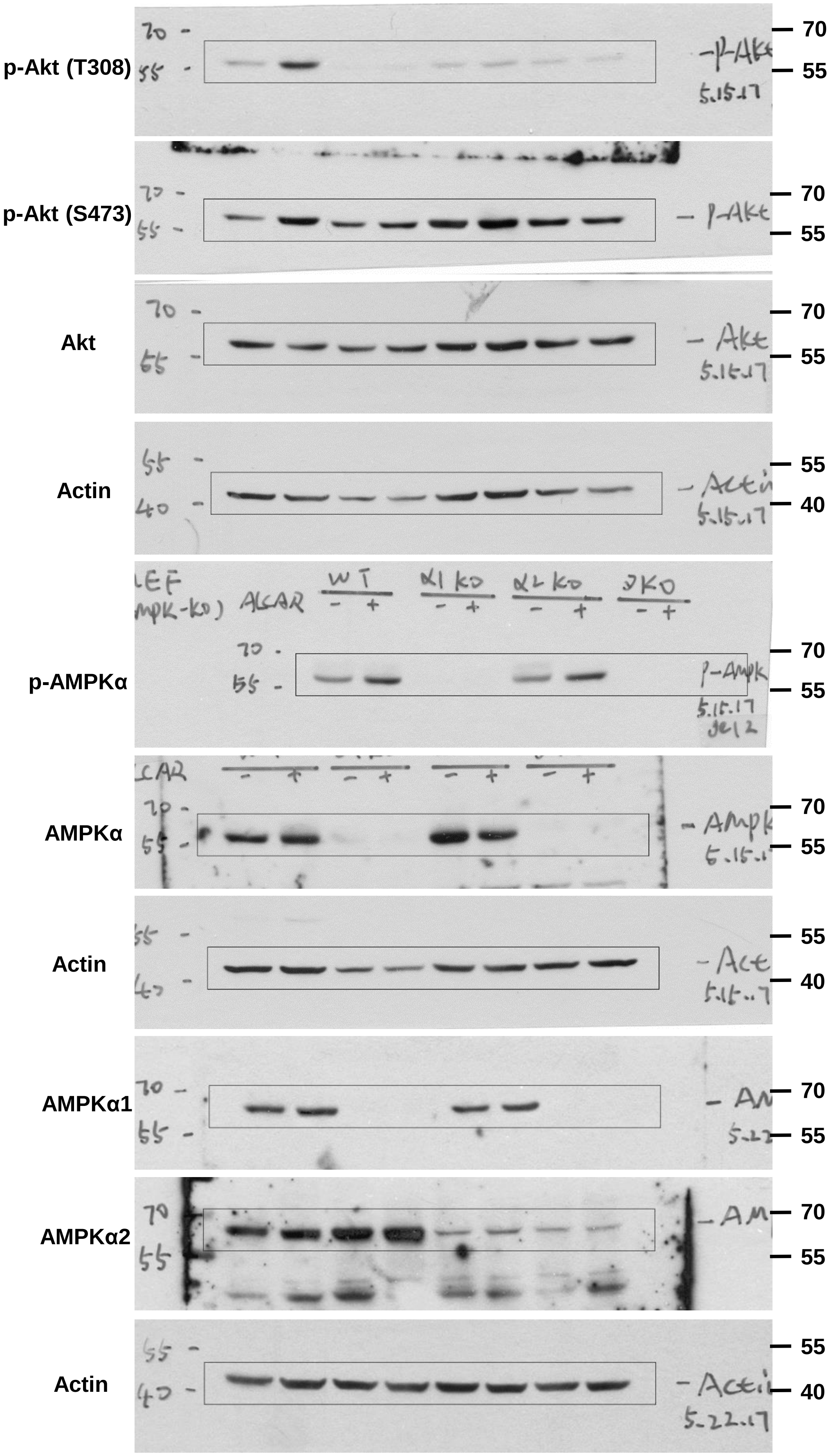

Supplementary Figure 26. Uncropped Western blots for Figure 5G.

Figure 5H

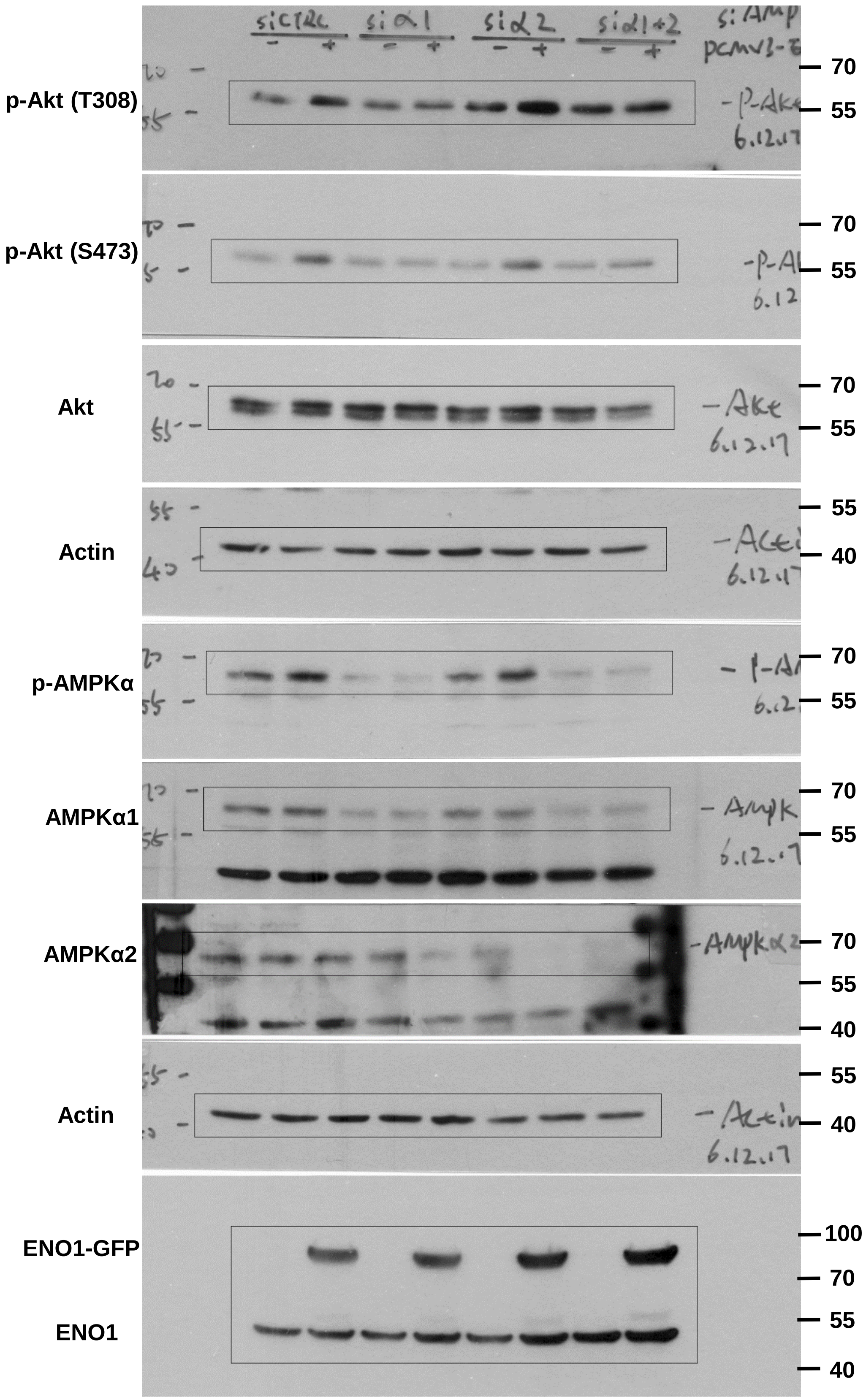

Supplementary Figure 27. Uncropped Western blots for Figure 5H.

SFig. 1A

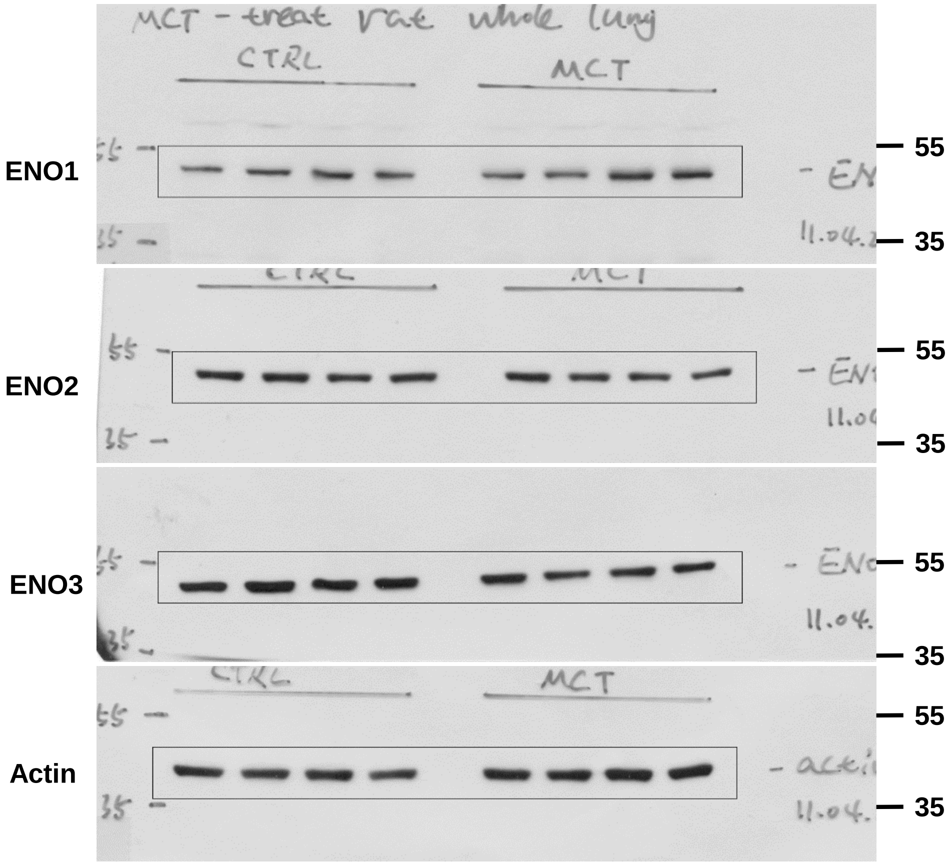

Supplementary Figure 28. Uncropped Western blots for Supplementary Figure 1A.

SFig. 6B

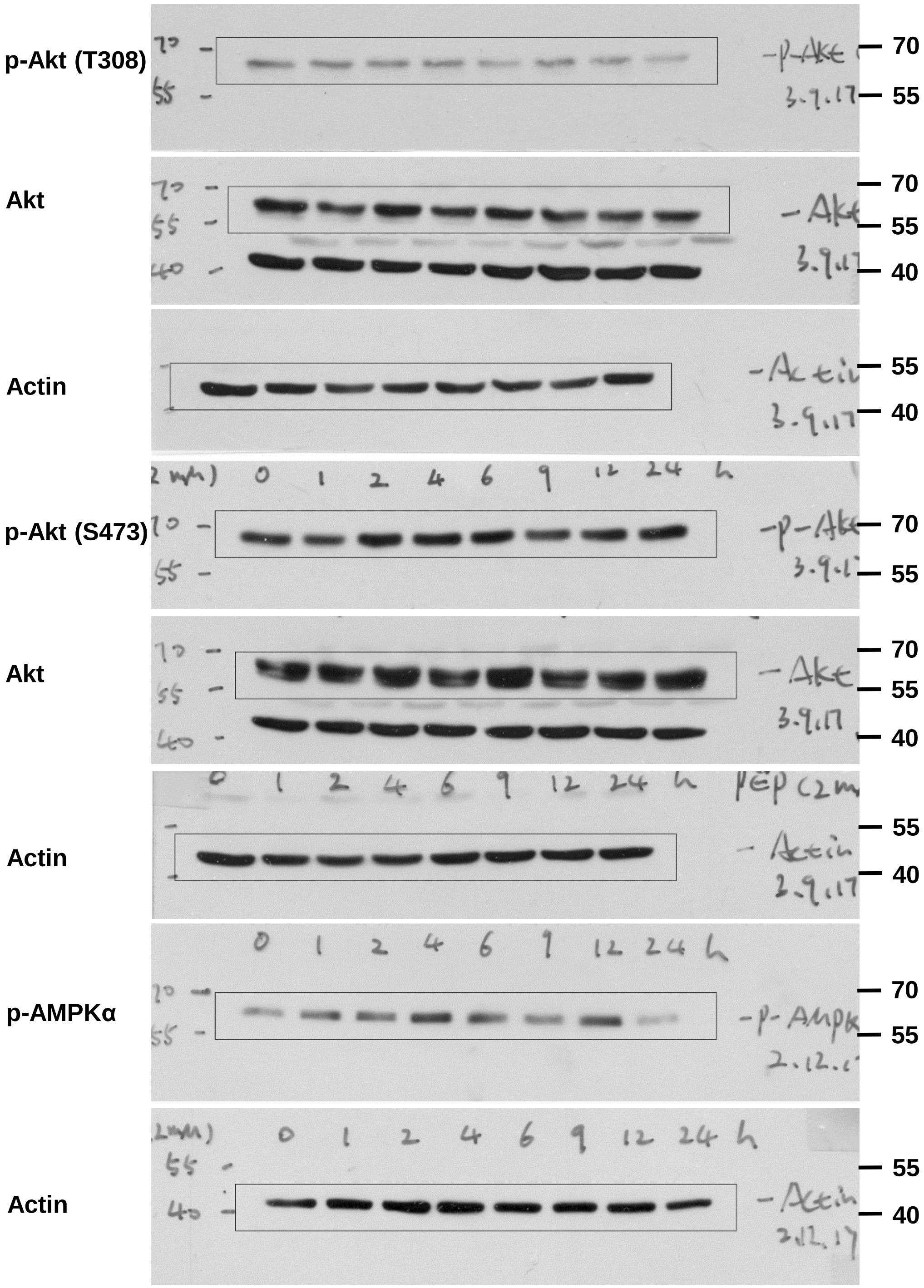

Supplementary Figure 29. Uncropped Western blots for Supplementary Figure 6B.

SFig. 7A

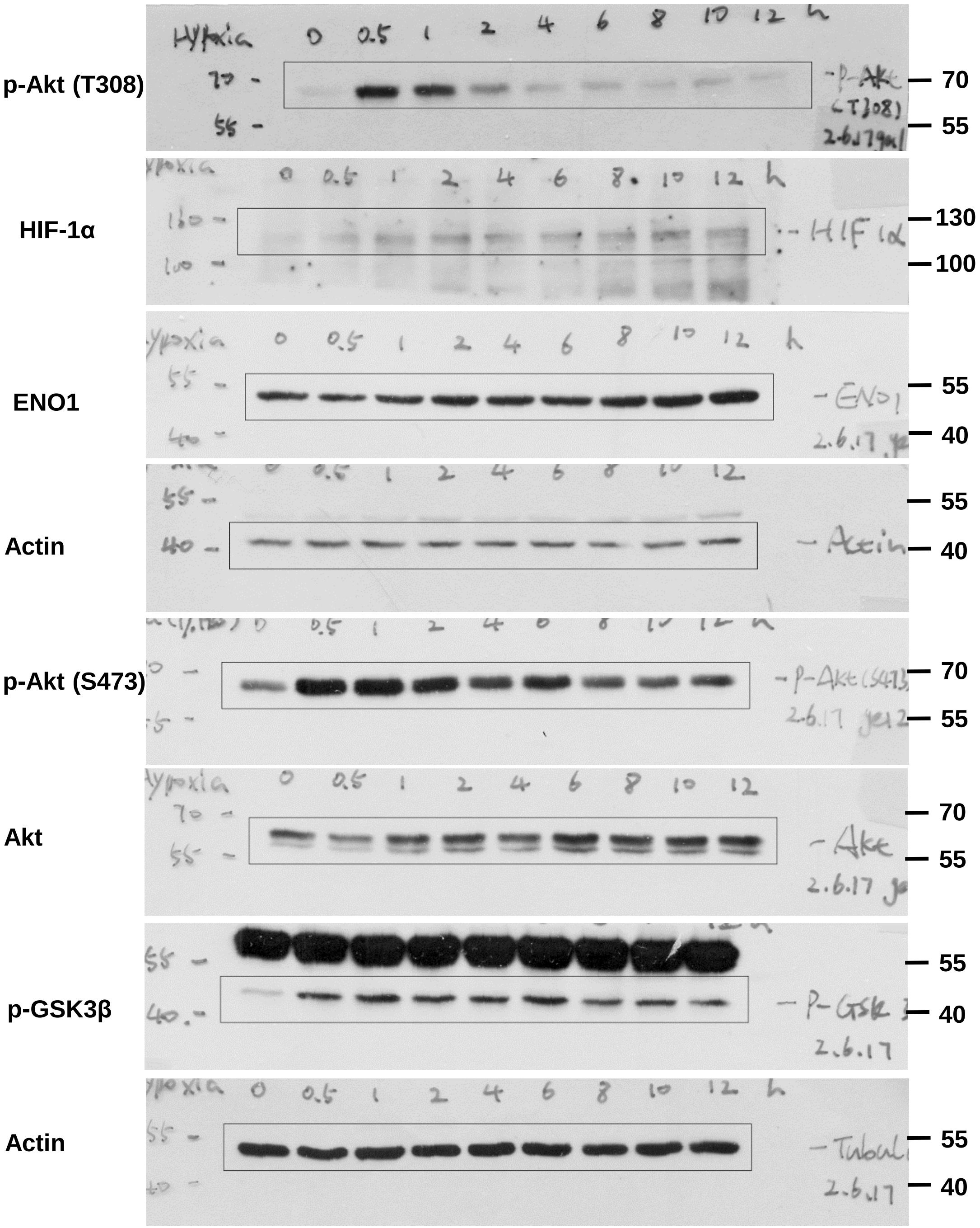

Supplementary Figure 30. Uncropped Western blots for Supplementary Figure 7A.

SFig. 7C

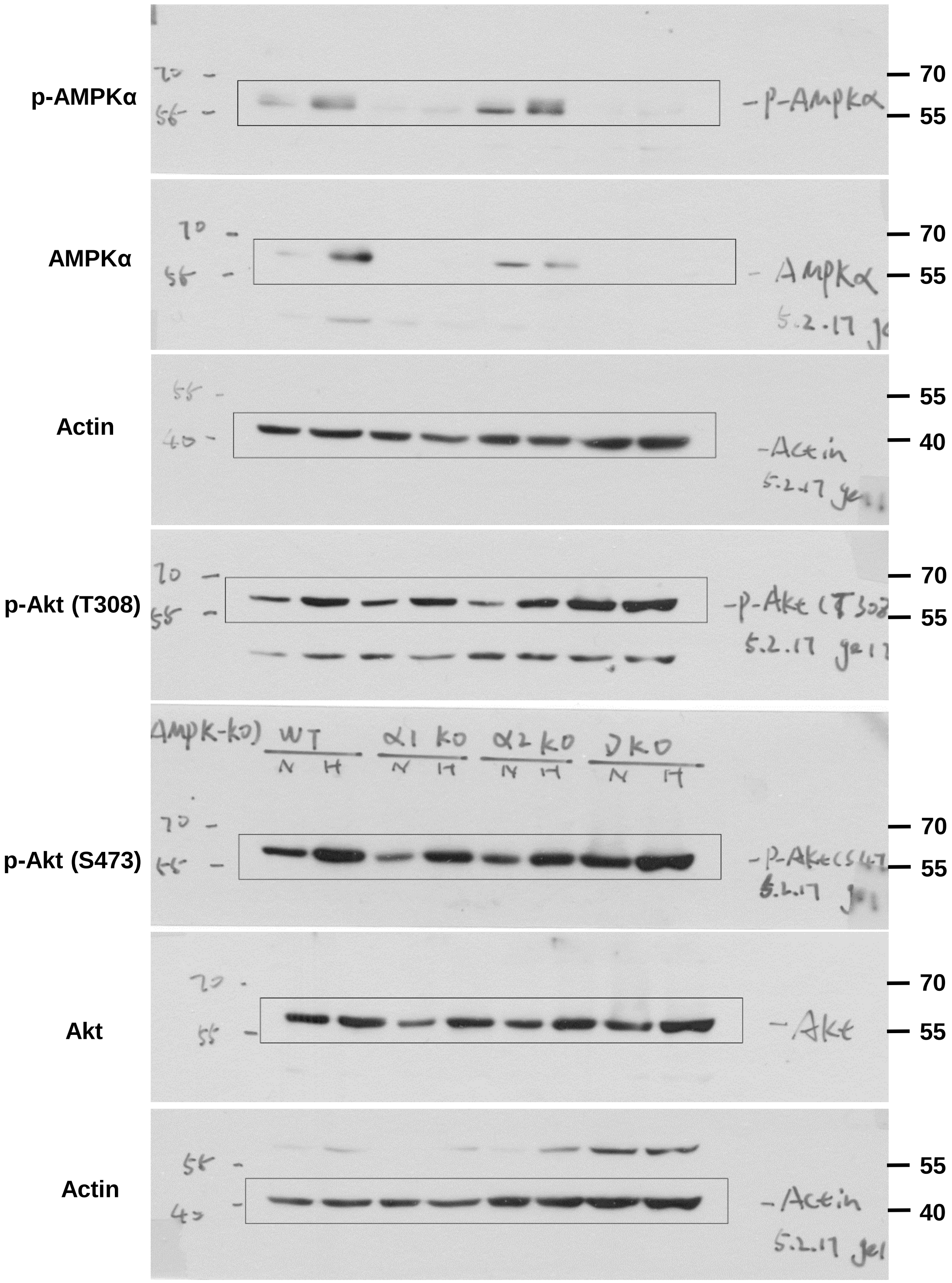

Supplementary Figure 31. Uncropped Western blots for Supplementary Figure 7C.

SFig. 8A

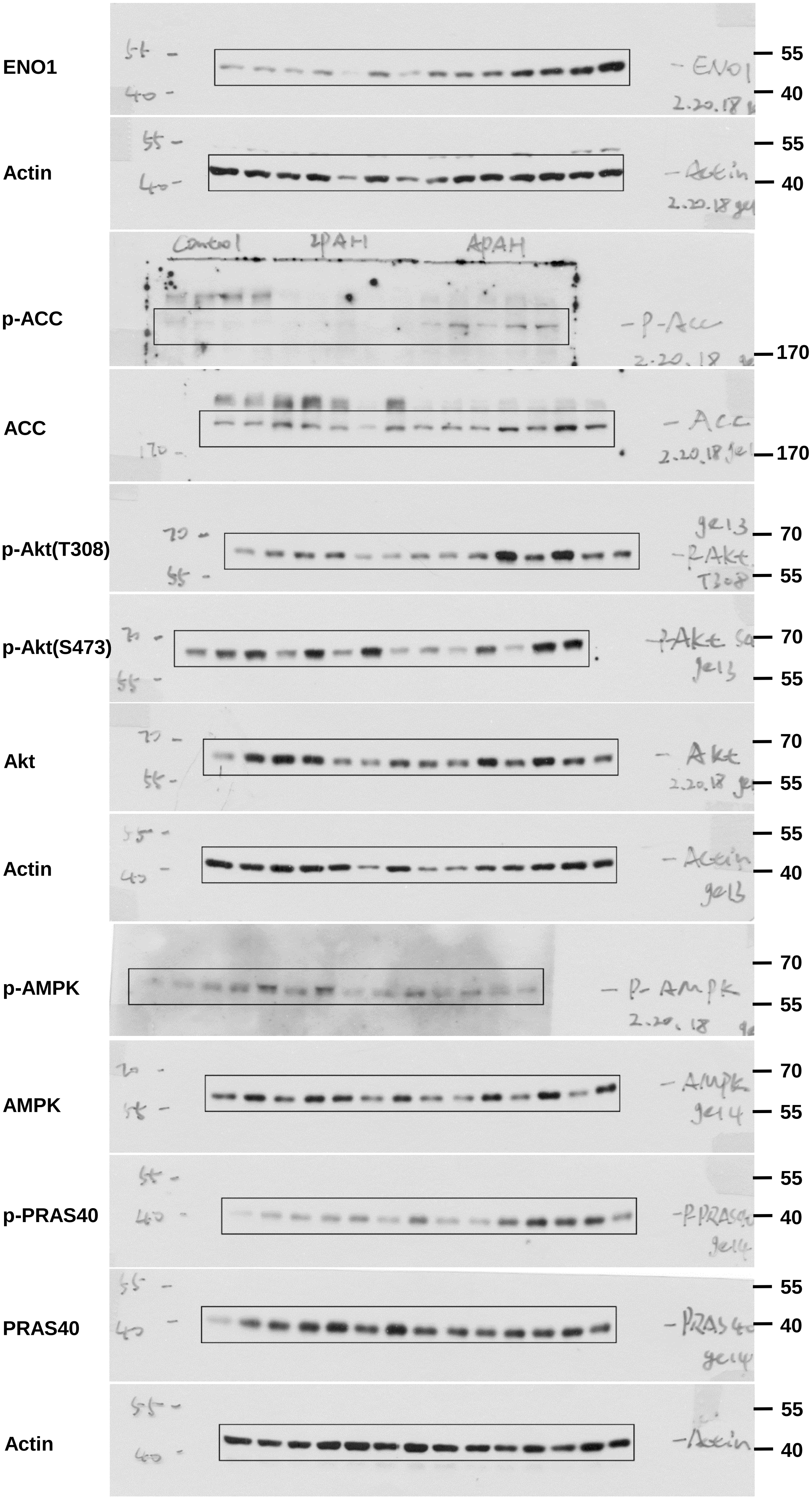

Supplementary Figure 32. Uncropped Western blots for Supplementary Figure 8A.

SFig. 13A

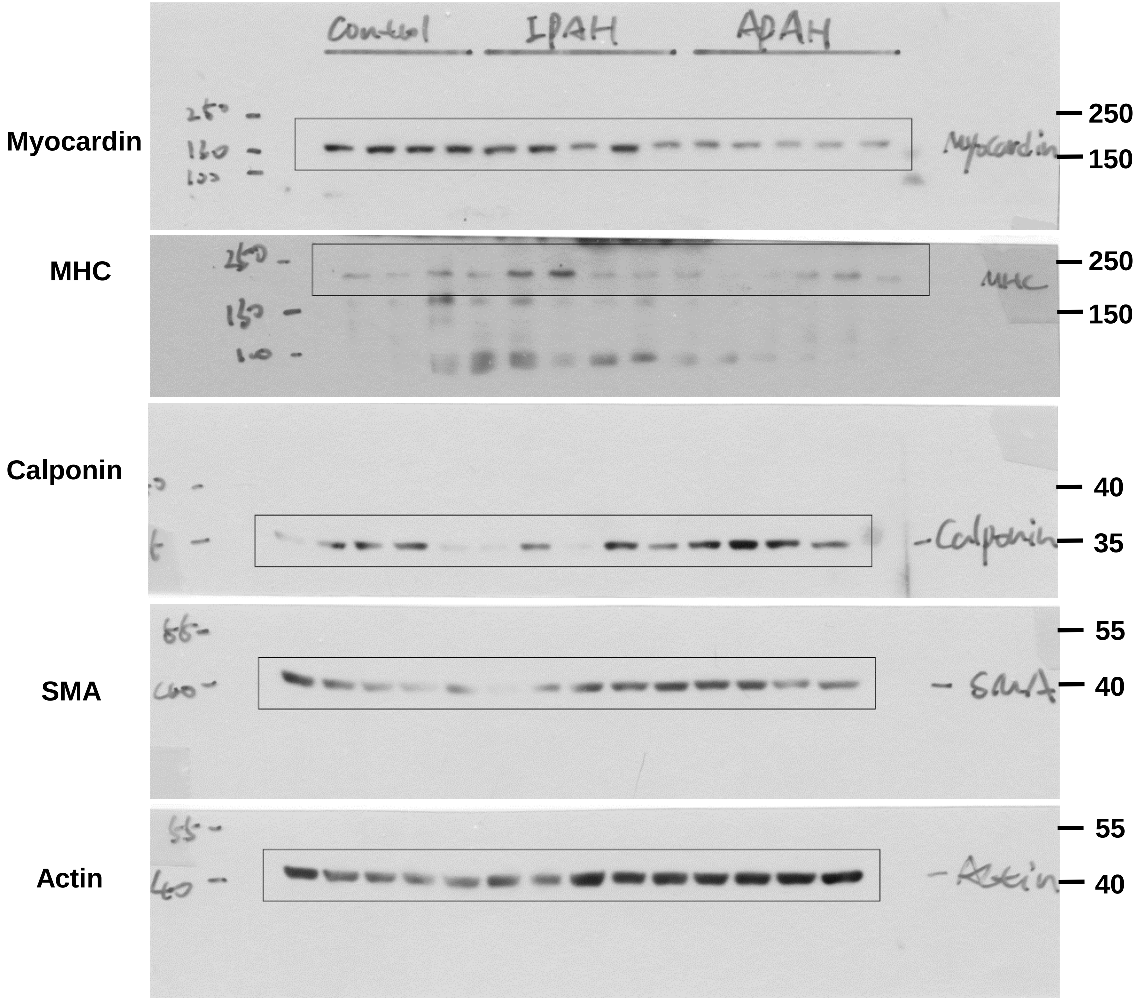

Supplementary Figure 33. Uncropped Western blots for Supplementary Figure 13A.

SFig. 15A

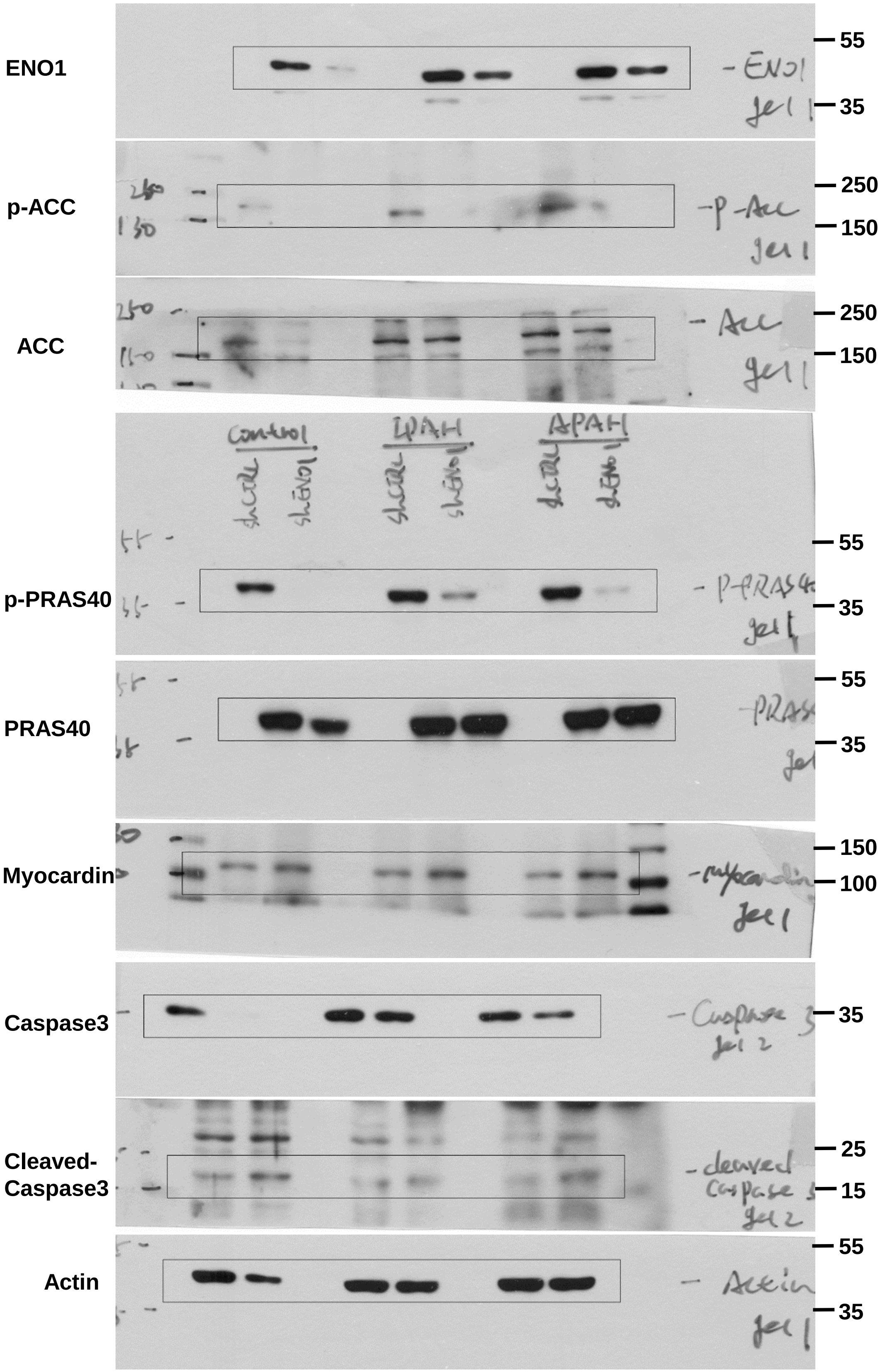

Supplementary Figure 34. Uncropped Western blots for Supplementary Figure 15A.

SFig. 15B

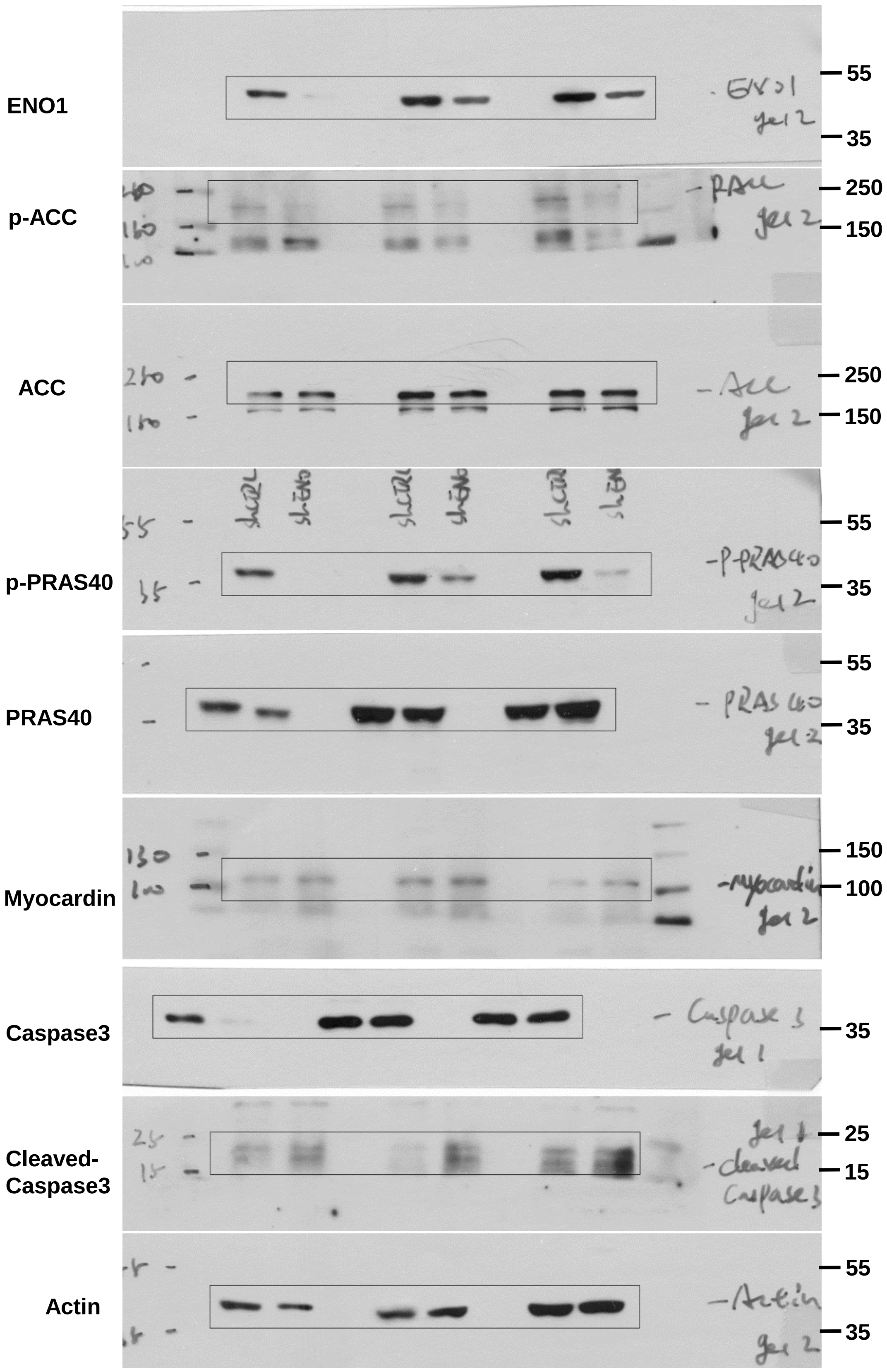

Supplementary Figure 35. Uncropped Western blots for Supplementary Figure 15B.

SFig. 15C

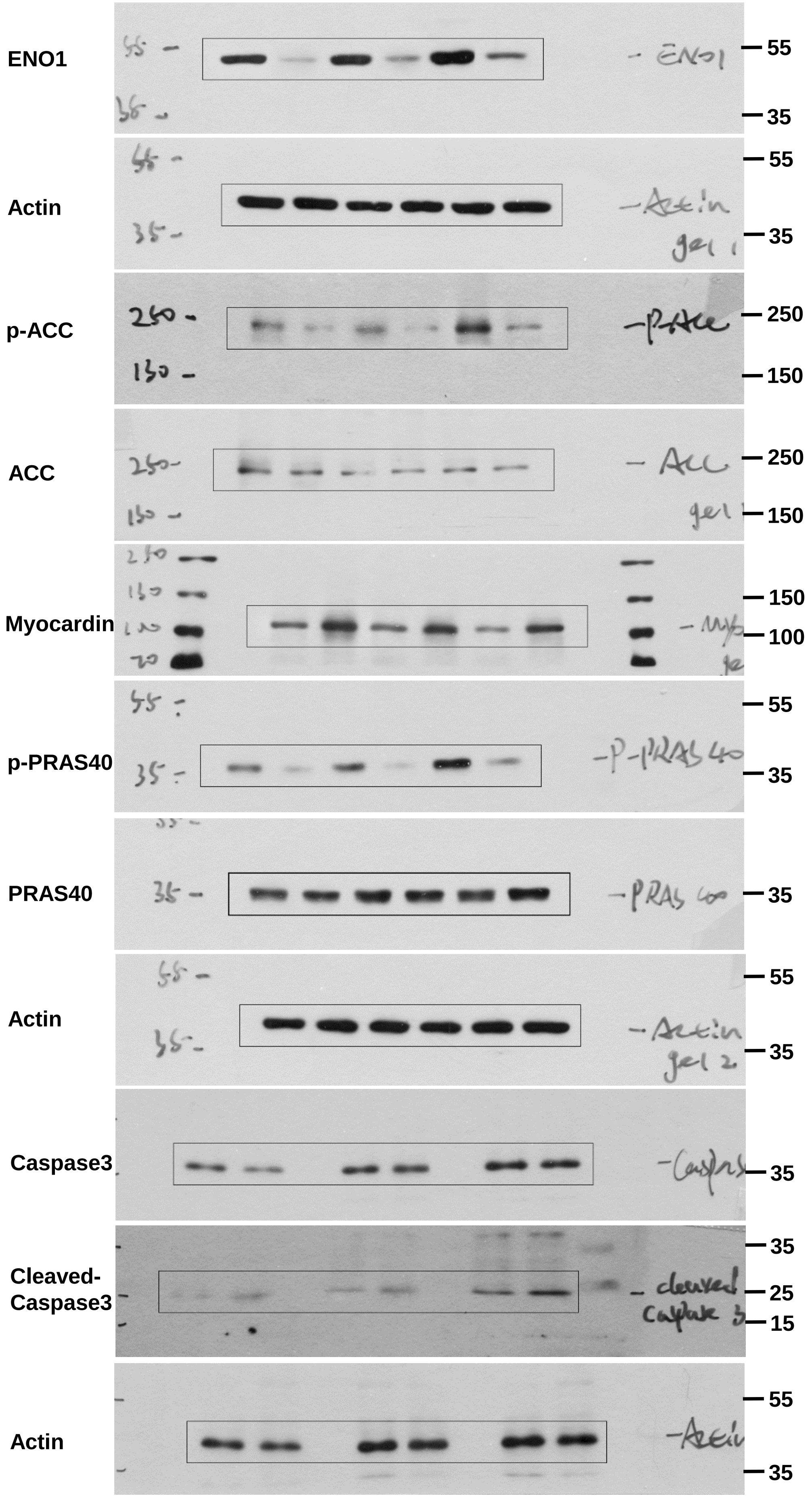

Supplementary Figure 36. Uncropped Western blots for Supplementary Figure 15C.

Supplementary Table 1. Baseline characteristics of PAH patients whose samples were obtained from PHBI.

	control subjects	IPAH	APAH *
N	5	6	6
Age, y	NA	38.2 (10.4)	36.5 (20.7)
Female/male	3/2	4/2	4/0
mPAP, mmHg	NA	52.8 (10.2)	55.6 (26.9)
PVR, wood units	NA	11.3 (3.8)	9.5 (2.5)
6MWD, m	NA	386.7 (71.4)	327.4 (177.8)
Race**	5W	2W/1A/3B	2W/1A/1U

Data are shown as mean (SD).
*: Information of 2 patients are unavailable.
**: W for White, B for Black or African American, A for Asian, and U for unknown.

Supplementary Table 2. qPCR primers used in this study

Primer ID	Sequence (5’-3’)
hRPL19-S	ATCATCCGCAAG CCTGTG
hRPL19-A	TGA CCTTCTCTG GCATTC G
mRPL19-S	AGC CTG TGA CTG TCC ATTC
mRPL19-A	ATC CTC ATC CTT CTC ATC CAG
rRPL19-S	AGC CTG TGA CTG TCC ATT C
rRPL19-A	ATC CTC ATC CTT CGC ATC C
hENO1 -S	ATCTCACAGTGACCAACCCA
hENO1-A	TGGTTGACTTTGAGCAGGAG
hENO2-S	CTGTGGTGGAGCAAGAGAAA
hENO2-A	TGGATTTGTTCTCAGTCCCA
hENO3-S	CCACGGGTATCTATGAGGCT
hENO3-A	CAGCCTTCAGGACTCCTTTC
rENO1-S	ATCCTTAGAATCGAGGAGGAGC
rENO1-A	TCTCCGGTCCATGCCTTACT
rENO2-S	GTACGGCAAGGATGCCACTA
rENO2-A	GCTCCAAAGCTTCGCTGTTC
rENO3-S	TGGAGAACAATGAGGCCCTG
rENO3-A	GCCACATCCATGCCAATCAC
mENO1-S	AGCGATCCTACTGCCAGAAAT
mENO1-A	GATCGACCTCAACAGTGGGA
mENO2-S	TCATGAGAATTGAGGAAGAGCTGG
mENO2-A	GTTCAGGCAAGCGGGGTT
mENO3-S	AAGGAAGGCTTTCCAGCTCCA
mENO3-A	AAGAGTGGACAGTTGATCCCTT
